# SERA-Net: Rethinking CNN Design for Brain Tumor Classification via Squeeze-and-Excite Attention and Residual Learning

**DOI:** 10.3390/bioengineering13091055

**Published:** 2026-09-11

**Authors:** Jyoti Ranjan Sahoo, Surendra Kumar Nanda, Ganapati Panda, Ashutosh Soni, Jayanti Rout, Manob Jyoti Saikia

**Affiliations:** 1 Biomedical Sensors & Systems Lab, University of Memphis, Memphis, TN 38152, USA; 2 Department of Computer Science and Engineering, C. V. Raman Global University, Bhubaneswar 752054, Odisha, India; 3 Electrical and Computer Engineering Department, University of Memphis, Memphis, TN 38152, USA

**Keywords:** brain tumor, magnetic resonance imaging, deep learning, CNN, pretrained model

## Abstract

A brain tumor is a life-threatening disease that carries a high mortality burden. It can be treated if detected in the early stages. Manual analysis by a radiologist using Magnetic Resonance Imaging (MRI) is effective but slow and subject to inter-observer variability. In order to automate it, various deep learning models, especially Convolutional Neural Networks (CNNs), have been widely used. However, most of them rely on frozen, ImageNet-based pretrained backbones, where only a small classification head is updated during training. This leaves a large segment of parameters unable to adapt to the domain shift between natural images and MRI scans. This limits how far such models can be modified or improved further. This makes the model highly parameter-centric and unsuitable for development. In addition, most studies fail to provide insights on architectural choices, which inhibits reproduction and modifications. In order to alleviate these issues, this study presents SERA-Net, a convolutional architecture for brain tumor classification using MRI scans. It is trained from scratch and quantifies the performance of each model using an incremental ablation study. It consists of four blocks comprising different components, such as Squeeze-and-Excite channel attention, residual connections, a wider classification head, etc. Rather than proposing new operations, the contribution lies in the systematic, ablation-driven combination and empirical validation of established architectural components for this specific task. The model was trained and evaluated on a publicly available MRI dataset. With just ≈5 million parameters, it achieved an accuracy of 95.44%, an F1-score of 0.953, and various near-ideal parameters. It was observed to outperform six pretrained CNN baselines by more than 5–10%, while using substantially fewer parameters. Various other analyses indicate the efficacy of the proposed approach in detecting brain tumors. The proposed approach provides a scalable design that can be adapted to different domains with limited modifications.

## 1. Introduction

Brain Tumors (BTs) are one of the most lethal forms of cancer. They involve the development of an abnormal mass of cancerous cells. According to one study, almost 20 million new cases, followed by nearly 6 million deaths, were estimated to have occurred in the United States alone in 2025 [1]. These figures show the gravity of the issue. In addition, according to the Global Cancer Observatory and various other organizations, brain and Central Nervous System (CNS) cancers rank 19th among various diseases in terms of new cases and 11th in terms of mortality [2]. On-time detection as well as BT Classification (BTC) of tumors can lead to planned treatment, which can help in saving patients.

Magnetic Resonance Imaging (MRI) is one of the widely used methods of imaging for BTs. Its non-ionizing property makes it more suitable than X-rays for patients that require multiple follow-ups. Medical practitioners also use it due to its ability to distinguish between normal brain tissue, tumors, swelling, and fluid much better than X-rays or Computed Tomography (CT) scans. Manual interpretation of MRI scans by medical experts is a leading approach to detection. However, it is slow relative to the volume of scans generated. In addition, it is also subject to inter-observer disagreement on borderline cases. This motivated the use of Artificial Intelligence (AI) models [3]. Although Machine Learning (ML) approaches were used initially to aid in automated classification, they relied on handcrafted texture features like wavelet descriptors. This means that they only capture the patterns a human designer thought to encode in advance. As a result, Deep Learning (DL) approaches came into play as a better alternative. Models like Convolutional Neural Networks (CNNs) can automate extraction of features from the input image [4]. They also support a range of Pretrained Models (PTMs) that are already trained on large datasets like ImageNet.

Even within these CNN-based approaches, there are various gaps that limit how much confidence can be placed in the reported results. A large number of research studies use a frozen, ImageNet-based PTM with a small classification head fine-tuned on the target dataset. However, few studies compare this Transfer Learning (TL)-based approach against models that are built from scratch. In this case, the textures of MRI differ substantially as the PTMs were trained on natural images. Fine-tuning more layers of a PTM is technically possible. However, it comes at the cost of increased risk of overfitting on small medical datasets, along with a higher training and memory budget. As a result, most works in this space keep the backbone frozen and rely on the pre-learned ImageNet features to transfer as-is. In addition, such models are highly parameter-centric in nature, which increases the model size. This issue becomes even more grave when different PTMs or models are ensembled [5]. Although this approach minimizes architectural dependencies of a single model, it increases architectural complexity and inference time. Even though various researchers use models built from scratch, they under-explain the role of each architectural component. This inhibits ease in reproduction of such designs in practice.

In order to alleviate these issues, this work presents a BT detection model using a convolutional model, built from scratch. The proposed design is a result of an incremental-based ablation study. It consists of four blocks and a wider classification head, along with a Squeeze-and-Excite (SE) channel attention, residual connections, Global Average Pooling (GAP), etc. In order to manage the Learning Rate (LR) adaptively, a Cosine Annealing LR Scheduler (CALRS) was used. It was trained and evaluated on a publicly available MRI dataset. With just ≈5 million parameters, it achieved a test accuracy of 95.44% and an F1-score of 0.953, along with various near-ideal performance metrics. It achieved a Brier loss of near zero that indicates confidence while making predictions. Various other simulation-based experiments and analyses highlight the efficacy of the proposed approach in the classification of BTs.

The remaining portions of the paper are classified as follows: Section 2 presents the outline of various research works. Section 3 and Section 4 present the methodology of the proposed output and quantification of performance, respectively. Section 5 concludes the work after discussing various potential works.

## 2. Related Works

BTC is an emerging research topic that involves various open challenges, like the development of lightweight models without loss in classification performance, accurate detection, reliable performance, etc. In order to achieve effective classification performance, researchers adopted various approaches, including ML, DL, You Look Only Once (YOLO), PTMs, ensemble models, pruning, and quantum-enhanced algorithms [6,7,8,9]. Different frequency-domain transforms and metaheuristic algorithms are also extensively used for improving overall performance [10,11,12].

Yahya et al. [13] used a pruning-based EfficientNetV2B3 model to extract relevant features for BTs. Different metaheuristic-based feature selection techniques are used to improve decision-making. The model is combined with different ML models for classification. Similarly, Uniyal et al. [14] presented a random forest-based feature selection approach with a generalized regression neural network. Particle swarm optimization was integrated for improved performance. Along the same line of research, Panda et al. [15] developed a radial-basis Gaussian function neural network combined with a grey wolf optimizer for BTC.

Puligurti et al. [16] developed a multilayer BT detection approach that combines a multilayer extreme learning machine (fast learning capacity) and a progressive adjacent-layer coordination symmetric cascade model (identifies boundaries of BTs). It consists of an autoencoder-based module for deep feature extraction and a tactical unit algorithm for stability. Similarly, Wessam et al. [17] combined synthetic data generation for augmentation, segmentation, and dual PTM-based classification. It involves a combination of ResNet50 and VGG16 for classification. Complementing this dual-stream approach, Zada et al. [18] used a combination of Vision Transformer (ViT) and CNN for BTC. CNN is used for identification of local features, while the latter analyzes the entire image using self-attention.

Similarly, Hasan et al. [19] presented a strip-style pooling attention network that utilizes the strength of both channel attention and spatial attention modules. It utilizes strip pooling and style pooling to capture long-range spatial dependencies as well as rich texture details at the same time. Following a similar line of work, Rajmohana et al. [20] developed a hybrid approach that uses a conventional graph CNN and a quantum-enhanced graph CNN model for BTC. The latter aids in improving the performance of the conventional model.

Abdusalomov et al. [21] used YOLOV7 for BTC. Similarly, Taha et al. [22] used fine-tuned YOLO models, specifically, YOLOV8 and YOLOV11, for BTC. Complementing this approach, Chen et al. [23] presented YOLO-LS for BT detection that involves the integration of ShuffleNet V1 for feature extraction and C3k2-PoolingFormer to improve multi-scale feature fusion.

From the above-discussed works, it can be observed that there are various approaches that utilize different PTMs for classification. In addition, a large number of works involve the utilization of dual-stream or ensemble approaches for classification. Although such approaches improve classification performance, they tend to make models complex in nature. This results in an increase in parameter count and thus an increase in model size. Drawing inspiration from [13], where threshold-based pruning is used to reduce the model size with the least effect on performance, this study presents a CNN-based model built from scratch for BTC with just ≈5 million parameters. It consists of carefully selected architectural components and design analogies that help in achieving overall improvement in BTC performance.

## 3. Methodology

**Figure 1:**
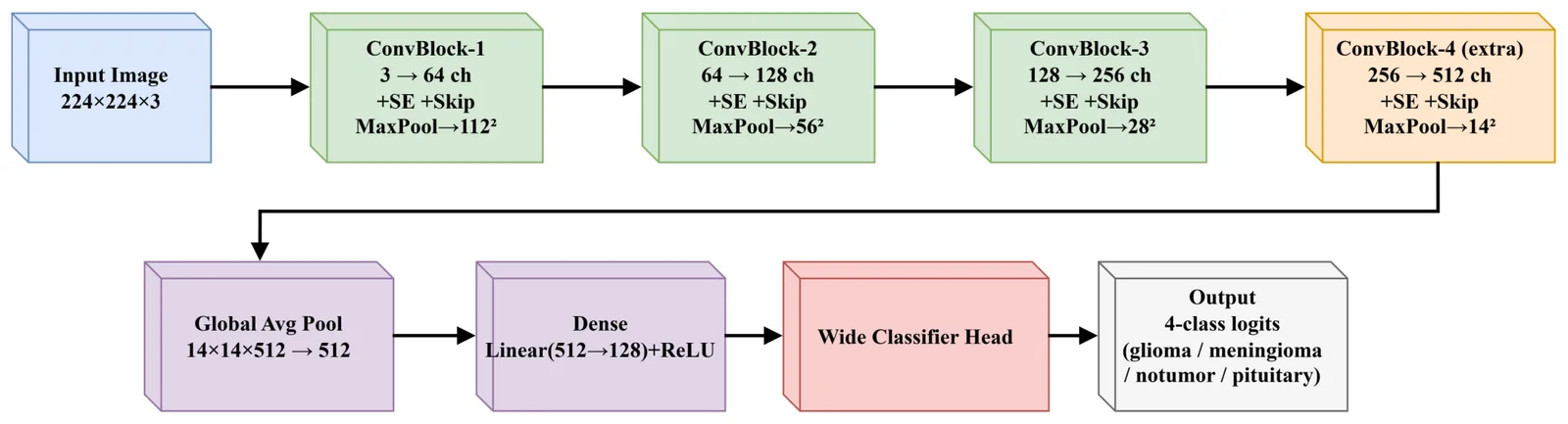
Overall working strategy of the proposed architecture.

The proposed system classifies brain MRI scans into four categories: glioma, meningioma, pituitary tumor, and no-tumor. The overall pipeline consists of three stages: (i) preprocessing and augmentation of the input MRI images, (ii) a CNN that extracts hierarchical spatial features and produces class probabilities (Figure 1), and (iii) a training procedure that optimizes the network parameters using a fixed configuration of optimizer, LR schedule, and regularization. The final model used for reporting results combines a deeper convolutional backbone, channel attention, residual connections, and a wider, more strongly regularized classification head.

### 3.1. Dataset and Preprocessing

The model is trained and evaluated on a publicly available BT MRI dataset [24]. No personally identifiable information was present in the dataset. The images are pre-split into train and test splits, including 5600 and 1600 images, respectively. Each split contains an equal number of images per class. This enables efficient training of the model, inhibiting the inherent risk of bias towards a specific class due to class imbalance. This dataset is a combination of three widely used datasets: the figshare brain MRI dataset, the SARTAJ dataset, and the Br35H dataset. This dataset was specifically selected, as it provides a wide range of images, curated from different MRI acquisition settings, imaging centers, etc. This enables an effective training strategy rather than relying on a homogeneous dataset.

Every image is resized to 224×224 to maintain a fixed spatial resolution for input images. The intensities of pixels are normalized channel-wise using the ImageNet mean (0.485, 0.456, 0.406) and standard deviation (0.229, 0.224, 0.225). This normalization keeps the input distribution close to what convolutional filters expect. Using the pretrained weights also stabilizes activations in the early layers for better convergence, even if the model is trained from scratch.

In order to make the model robust to common image perturbations, various augmentation techniques were applied to the training set. They included random horizontal flipping, random rotation up to $15^{\circ }$, and color jittering of brightness and contrast (both varied by up to ±20%). These transformations were selected as they represent natural changes in images due to variations in patient positioning, scanner contrast settings, differences in orientation between MRI slices, etc. On the other hand, the test set was only resized and normalized. No additional augmentation was applied in order to preserve its original characteristics. Since this dataset merges images from three separate sources, it was checked for any file-level duplication. This test ensures that there is no accidental file-level overlap between the training and test splits. Each image in both splits was hashed using MD5. The resulting hashes were compared for both splits to identify byte-identical files. No duplicate images were found between the train and test sets.

### 3.2. Convolutional Block

The basic processing unit of the proposed model is a convolutional block that consists of two 3×3 convolutions. Each one is followed by Batch Normalization (BN) and a Rectifier Linear Unit (ReLU) activation, along with a 2×2 max-pooling operation at the end. Equation (1) presents the resultant output feature map.

(1) $$h=\mathrm{ReLU}\left(\mathrm{BN}({\mathrm{Conv}}_{3\times 3}\left(x\right))\right)$$

**Figure 2:**
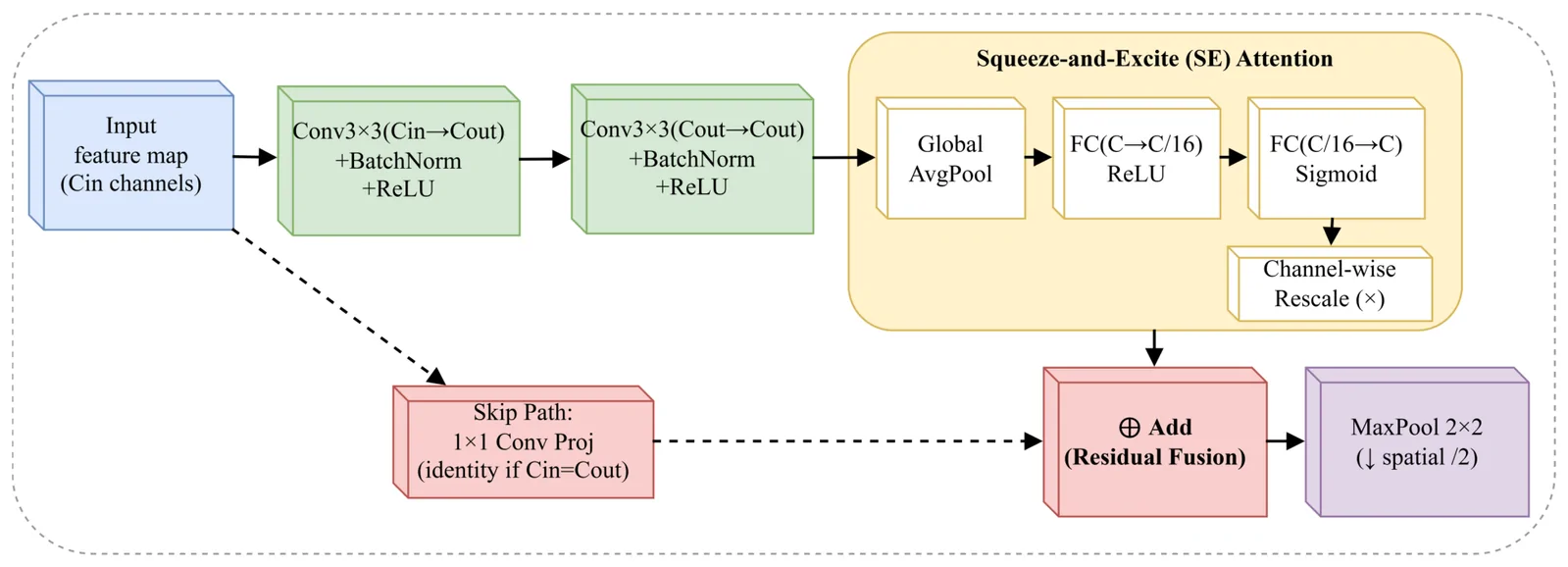
Architectural details of the ConvBlock.

Two convolutions per block allow the model to build a slightly larger, yet effective, feature extraction without needing a larger kernel. BN after each convolution keeps activations in a stable range. It also allows the model to use a higher LR without diverging. Max-pooling at the end of the block halves the spatial resolution. It reduces computation in later layers. It forces the model to summarize local features before passing them forward. The backbone stacks four such blocks with channel widths 3→64→128→256→512. The specific channel progression follows the common convention of doubling channel width whenever spatial resolution is halved. Figure 2 visualizes the architectural insights of the layer. This design balances feature extraction and computational cost as well as classification performance. This hierarchical design enables the model to learn abstract features, starting with low-level edges and textures, to high-level anatomical features relevant to BTC. In addition, increasing the number of channels with a decrease in spatial resolution improves the model’s representational capacity while maintaining computational efficiency. This keeps the amount of computation roughly balanced on blocks rather than concentrating it in the earliest or the deepest layers. The four-block depth was chosen as a middle ground. Fewer blocks would leave the final feature map too large and low-level to summarize tumor-level patterns effectively. On the other hand, additional blocks would shrink the 14×14 feature map further, at the risk of discarding the fine boundary and texture cues that distinguish tumor sub-types. This progression is also consistent with widely adopted CNN design strategies. As a result, this was retained as the backbone configuration for the proposed model.

### 3.3. Channel Attention (Squeeze-and-Excite)

Not every channel produced by a convolution is equally useful for a given input. In order to let the model focus on informative channels and suppress the less useful ones, an SE block is inserted after the two convolutions in every block. It first squeezes each channel’s spatial map into a single number using GAP using Equation (2). This channel descriptor passes through a small two-layer bottleneck. A reduction ratio of r=16, i.e., channels→channels/16→channels) with a ReLU in between and a sigmoid at the end, is used. This produces a channel weight $w_{c}\in \left(0,1\right)$. This weight rescales the feature map channel-wise, as defined in Equation (3). This reduction reduces the computational cost by keeping the number of extra parameters small while still letting the model learn which channels matter most for a given input. This also reduces inherent overfitting. The reduction ratio of 16 is an empirical/standard choice that is adopted, as it provides an effective balance between representational capacity and computational efficiency.

(2) $$z_{c}=\frac{1}{H\times W}\sum_{i=1}^{H}\sum_{j=1}^{W}x_{c,i,j}$$

(3) $${\tilde{x}}_{c}=w_{c}\cdot x_{c}$$

### 3.4. Residual Connections

Stacking multiple convolutional blocks can make optimization harder as gradients have to travel through more layers during backpropagation. In order to counter this, a residual connection is added inside each block whenever the input and output channel counts match. As a result, the block instead learns a residual function, as defined in Equation (4). f(x) represents the output of the two convolutions and the SE re-weighting. This shortcut provides gradients a direct path back to earlier layers. This helps training remain stable as the network gets deeper. When the input and output channels do not match, a 1×1 convolution projection is used to align dimensions before the addition. The block simply skips the residual addition and passes the convolution output forward if that alignment is not possible.

(4) y=f(x)+x

### 3.5. Global Average Pooling and Feature Embedding

After the final convolutional block, the feature map is reduced to a single vector per channel using GAP. It is followed by a linear layer with ReLU that converts the pooled 512-channel vector down to a 128-dimensional embedding. GAP is used instead of flattening the full feature map because it is far more parameter-efficient. It avoids large fully connected layers over a spatial map. This makes the model more tolerant to small spatial shifts in the location of the tumor.

### 3.6. Classification Head

**Figure 3:**
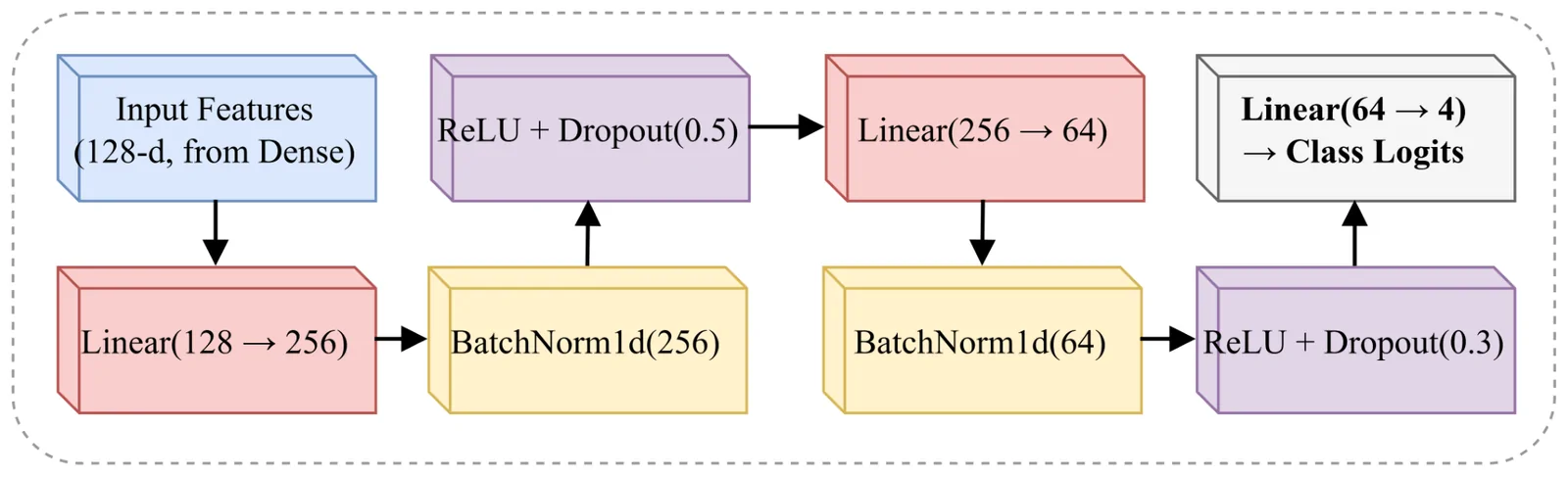
Visual interpretation of the regularized classifier head.

The 128-dimensional embedding is passed through a classifier head that converts it to the four output classes. The proposed model uses a wider head, with two hidden layers (128 → 256 → 64), as shown in Figure 3. BN is applied after each hidden layer. A dropout is applied between layers, i.e., 0.5 and 0.3 after the first and second hidden layers, before a final linear layer to the four class logits. The extra width compared to various baselines like 128 → 64 gives the classifier more capacity to separate the richer 512-channel features coming from the deeper backbones. The added BN and the two dropout layers were included to control overfitting, as a wider head also has more parameters that could memorize the training set. A softmax function is used to obtain the class probabilities from the logits.

Table 1 presents the layer-wise input/output shapes along with their parameter count of the proposed model (for an image resolution of 224×224×3). Dropout and activation layers that carry no parameters are omitted from the table for brevity.

### 3.7. Training Strategy

All convolutional layers that are followed by ReLU activations are initialized using Kaiming normal initialization. This preserves the variance of the feature representations in layers. This provides stable gradient propagation and enhances convergence during training. BN layers are initialized with weight 1 and bias 0. They preserve normalized feature distributions while allowing the model to learn appropriate scaling and shifting during optimization. The linear layers are initialized from a normal distribution (σ=0.01) with zero bias. This prevents unstable predictions or excessively large gradients at the beginning of training.

The model is trained using a multi-class cross-entropy loss between predicted class probabilities and ground-truth labels. Parameters are optimized using the Adam optimizer. It uses an LR of $1\times 10^{-3}$ and a weight decay of $1\times 10^{-4}$. Weight decay acts as an additional regularizer on top of dropout and BN. It penalizes large weight values and discourages overfitting. Instead of training the model randomly with different LRs with the aim of finding the best-suited one, a cosine-annealing LR scheduler is used for training. It is presented in Equation (5). Here, $\eta_{0}=1\times 10^{-3}$, $\eta_{\min}=1\times 10^{-6}$, and $T_{\max}$ is set to the total number of training epochs. As a result, the LR starts high and decays smoothly to a very small value by the end of training. This allows the model to make large updates early on and fine-tune more gently as training progresses. The best stage of the model was saved using the model checkpoint. After every epoch, the model is evaluated on the test set, and the checkpoint with the best accuracy is kept for final reporting rather than using the last epoch. This avoids penalizing a model that could overfit in its final epochs. Reporting the final-epoch weights would make the main result dependent on the last few optimizer updates. These updates may shift the decision boundary and may not reflect the model’s best performance. This is especially relevant when the LR is high, as performance can vary considerably between epochs. Selecting the best-performing checkpoint is a standard practice similar to early stopping. This approach avoids penalizing the model for a small performance drop during the final epochs of a fixed training schedule.

(5) $$\eta_{t}=\eta_{\min}+\frac{1}{2}\left(\eta_{0}-\eta_{\min}\right)\left(1+\cos\left(\frac{t}{T_{\max}}\pi \right)\right)$$

## 4. Results and Discussion

Various simulation-based experiments using multiple types of metrics are used to quantify the performance of the proposed approach.

### 4.1. Experimental Setup

All the training and experiments are conducted in a Kaggle Notebook environment with a single NVIDIA Tesla P100 GPU that includes 16 GB VRAM. This setup was selected due to its wide availability and balanced computational performance. Python3 and its various libraries were used for implementation. The model is trained for 100 epochs, with a batch size of 32. The training set is shuffled at every epoch. On the other hand, the test set was kept in a fixed order for consistent evaluation. A random seed of 42 was used in the approach to make it reproducible in nature. This seed controls the shuffling of the training set into mini-batches, weight initialization, selection of random augmentation parameters per image per epoch, etc.

### 4.2. Performance of the Proposed Model

**Figure 4:**
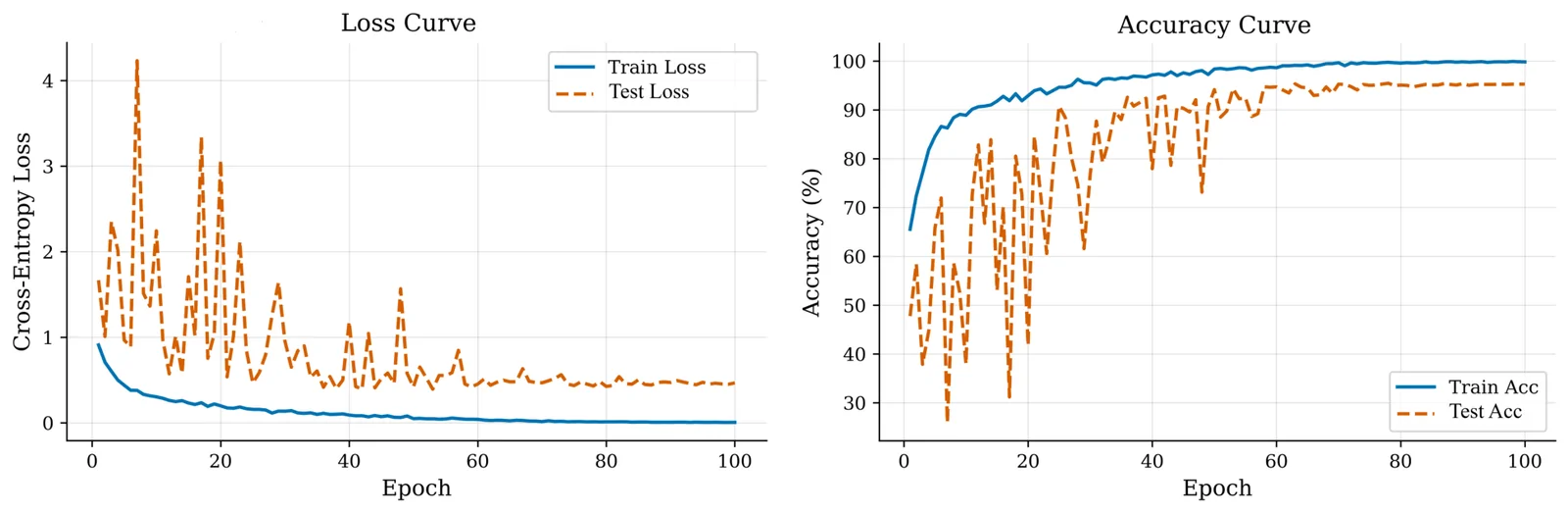
Trends in the loss and accuracy curves for the proposed model.

The trends in training and test metrics throughout the training of the proposed model are depicted in Figure 4. It lasted for the entire 100 epochs. Fluctuations were observed in the test curve, mostly in epochs 5–45. The CALRS starts at $1\times 10^{-3}$ and decays only gradually toward $1\times 10^{-6}$ over 100 epochs. During the early-to-mid training stage, the LR is still comparatively high.

As a result, each optimizer step can shift the decision boundary enough to affect test accuracy by 10–20% between consecutive epochs. The architectural depth and capacity also play a role in it, which introduces more parameters that must co-adapt during training. As the LR continues to decay in later epochs, the magnitude of effect also shrinks substantially. It is also reflected in the curves that stabilize around 60–70 epochs.

**Figure 5:**
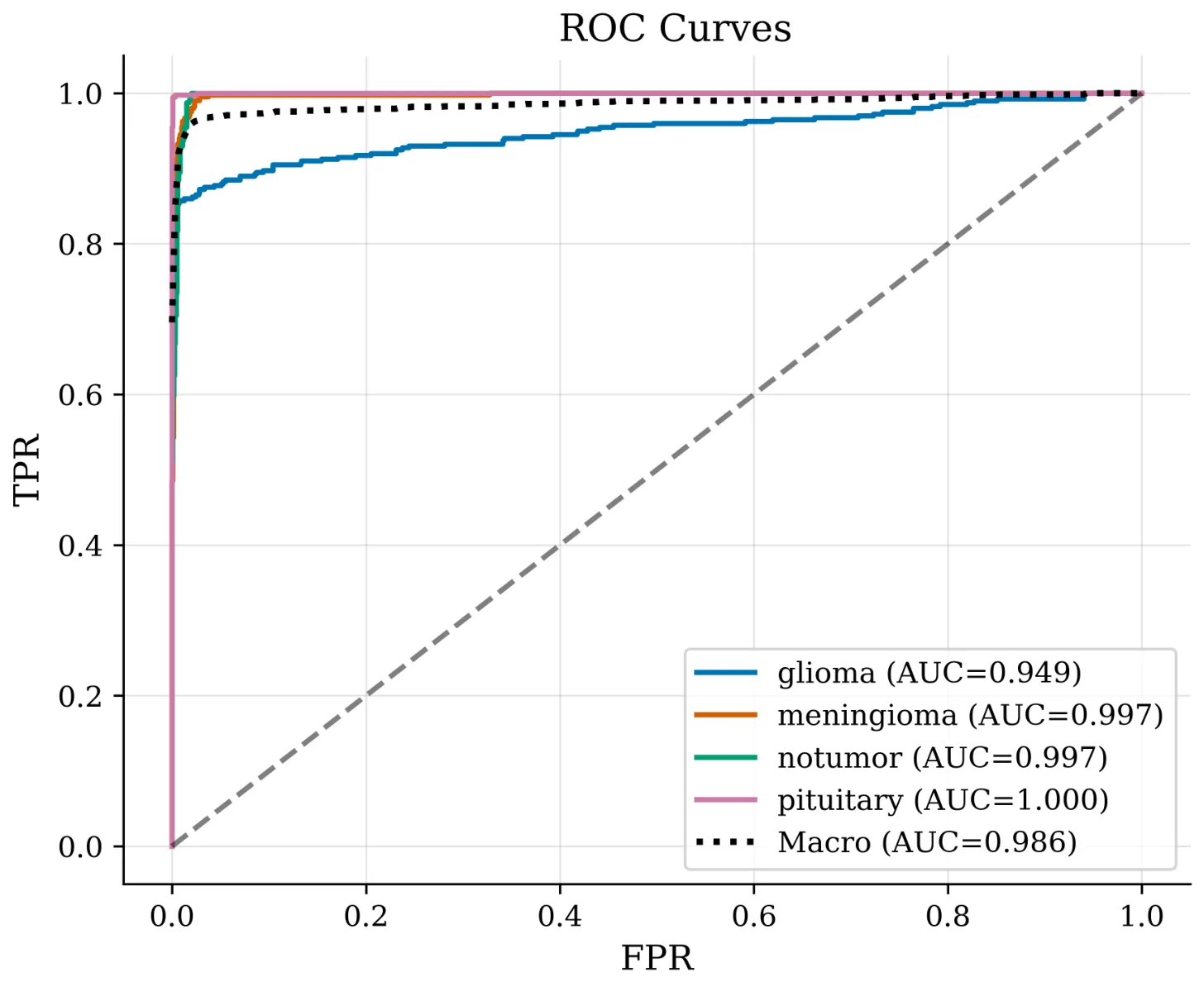
ROC curves for the proposed model.

**Figure 6:**
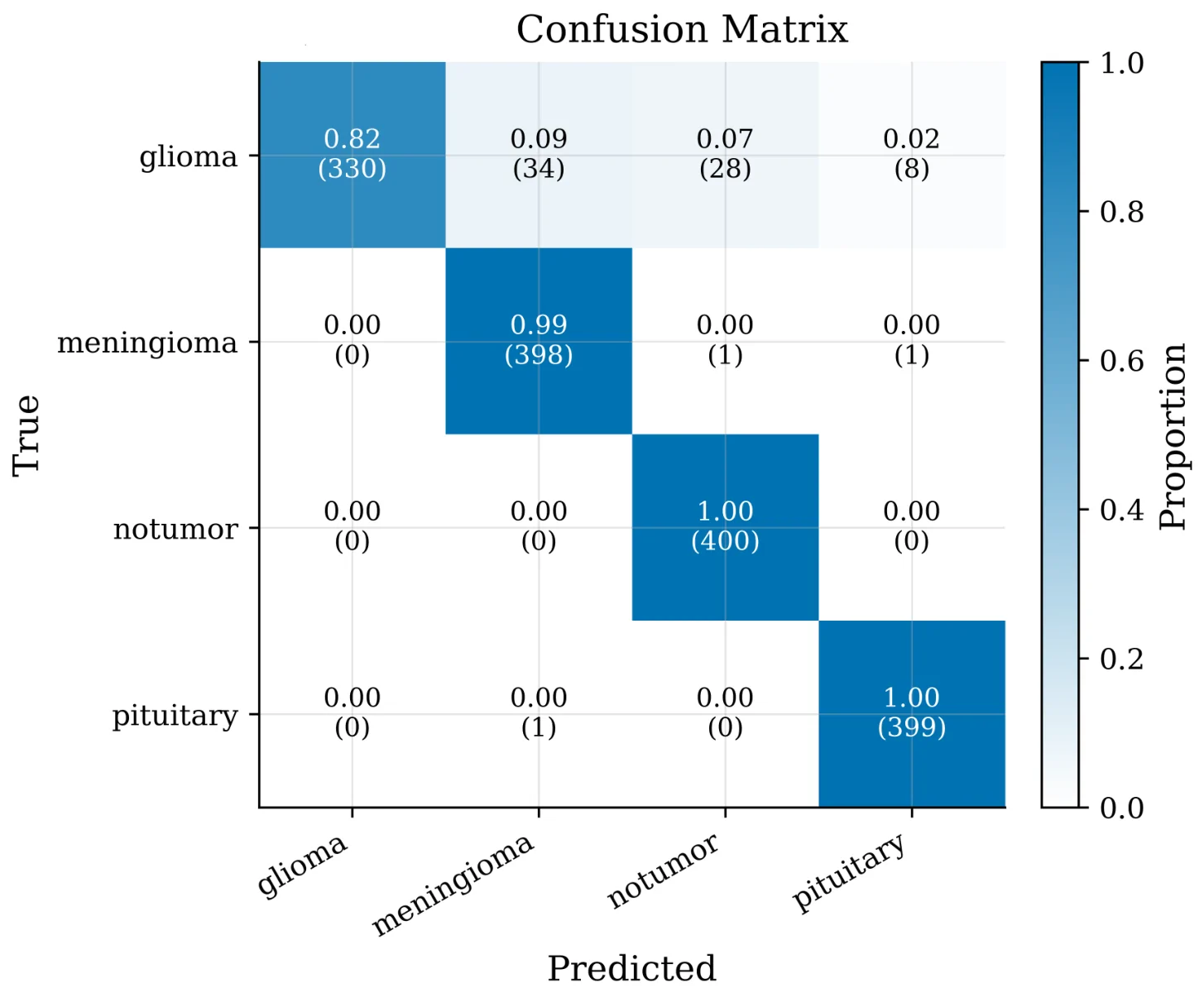
Normalized confusion matrix for the proposed model.

The model achieved a test accuracy of 95.44%, an F1-score of 0.953, and a Matthews Correlation Coefficient (MCC) of 0.940. The Area Under the Receiver Operating Characteristic Curve (AUC-ROC) and Area Under the Precision–Recall Curve (PR-AUC) of 0.986 and 0.980, respectively, indicate that the ranking of the predicted probabilities is close to ideal. Figure 5 visualizes the trends in ROC curves. The model usually predicts the correct class and assigns it higher confidence than the incorrect class. This matters when the probabilities are used for confidence thresholding, rather than just for selecting a label. This hypothesis is supported by a Brier score and log loss of 0.0214 and 0.426, respectively. The same is presented by the per-class metrics and confusion matrix in Table 2 and Figure 6.

The model demonstrates a well-balanced classification performance for all four classes. It achieved a macro-average precision of 0.957 and an F1-score of 0.953. The precision values are usually higher, which indicates that the model produces very few false-positive predictions. Recall is also near-ideal for most classes, reaching 0.995–1.0. This shows that the model produces few false-negative predictions, i.e., it rarely misses a tumor case that is actually present. No tumor and pituitary class achieved near-ideal metrics, as they both represent the most virtually distinct patterns. Although Glioma achieves a lower recall, its perfect precision indicates that every Glioma prediction made by the model is correct. This comes with a cost of missing a small number of cases rather than raising false alarms. This pattern is a result of the inherent visual complexity of the Glioma type compared with other types of BTs. Glioma sub-types are visually heterogeneous with infiltrative margins, compared to the more sharply bounded appearance of meningioma or the fixed location of pituitary tumors.

**Figure 7:**
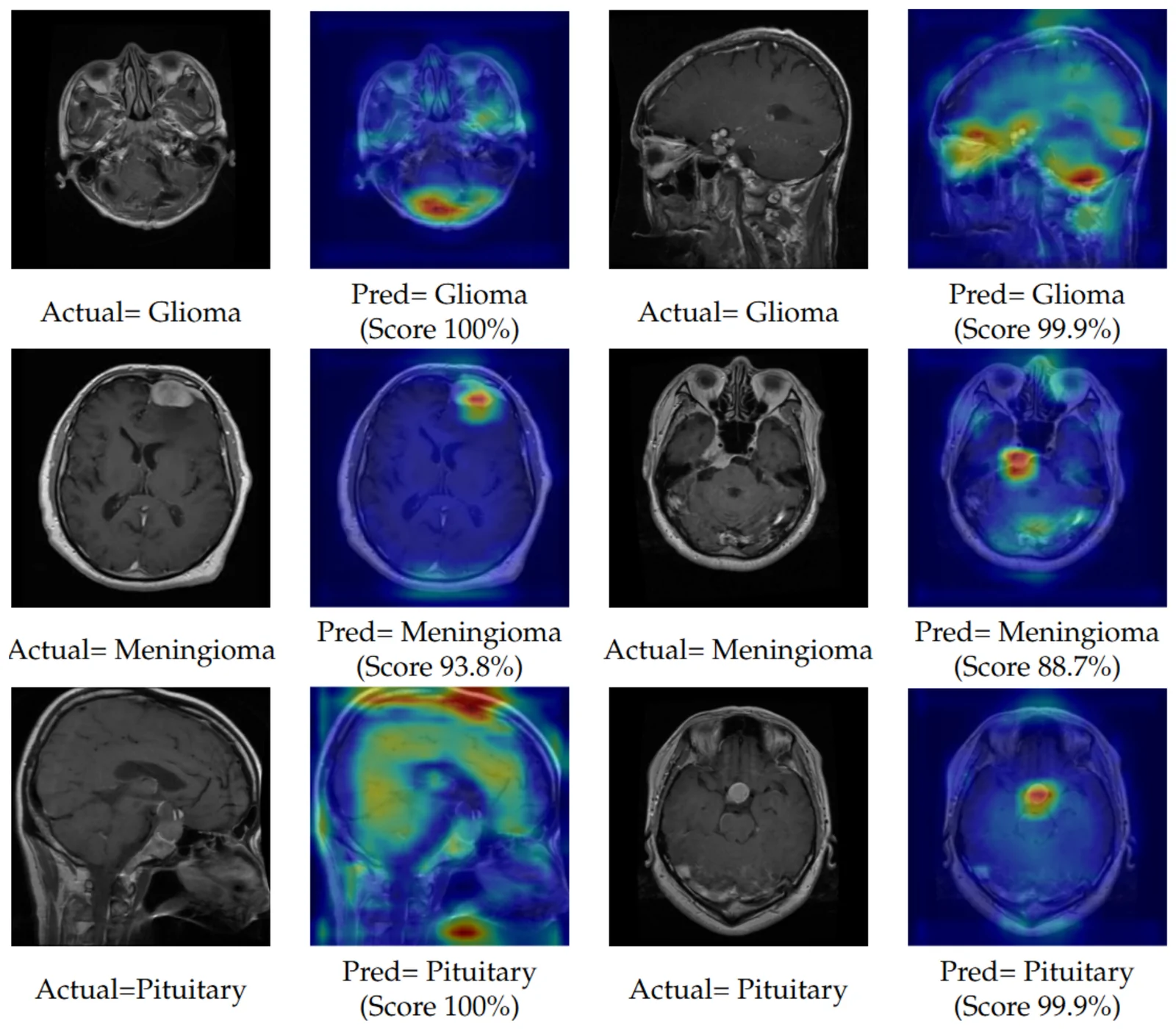
Explainability heatmap analysis of randomly selected images from the test set.

Figure 7 represents the explainability analysis using Grad-CAM++. Grad-CAM++ is a successor of Grad-CAM, which is widely used to inhibit the black-box nature of DL models. This analysis visualizes the concentrated regions in terms of heatmaps, which are responsible for making the particular decision. It was applied to the final convolutional block of the model. The target layer was the ReLU activation after the second convolution and batch normalization (conv2 → bn2) in the fourth (deepest) convolutional block. This layer was selected before the SE module and the final max-pooling operation. It was chosen because it is the deepest feature map that still maintains enough spatial resolution for meaningful localization. This strategy is a common choice in CAM-based explainability methods. For an input size of 224×224, the four convolutional blocks progressively reduce the spatial resolution through max-pooling. The target layer produces a 14×14 feature map with 512 channels. This feature map provides the information about activation and gradient required by Grad-CAM++. The activations and gradients are collected using PyTorch 2.13 hooks. A forward hook captures the activations. On the other hand, a full backward hook captures the gradients of the target class score with respect to these activations. A single backward pass is sufficient to obtain both. Grad-CAM++ was used over its predecessors, as it uses higher-order gradient information in order to calculate pixel-wise importance weights. In this implementation, these terms are approximated using powers of the first-order gradients. This avoids explicit higher-order differentiation. The resulting weights are used to calculate the importance of each feature-map channel. After this, the channel activations are combined using these weights. A ReLU operation keeps only regions that positively influence the prediction, and min-max normalization scales the resulting activation map to [0, 1]. The variations in confidence scores among different classes and samples indicate that the model is not spraying high confidence scores blindly. These results also align with the earlier-discussed classification performance. This analysis provides the medical experts a third eye for ease in judgment. These metrics indicate the efficacy of the proposed approach in making suitable decisions for multi-class BTC.

### 4.3. Ablation Study Analysis

In order to quantify the contribution of each architectural component, an incremental-based ablation study was conducted. It involved the addition of different components from a baseline while measuring the performance of each variant. Table 3 outlines the details of six ablated variants (V1–V6) along with their role, where V5 represents the proposed model. All the simulated variants were evaluated under an identical setup for a fair evaluation. A random seed of 42 was used for this experimentation.

**Figure 8:**
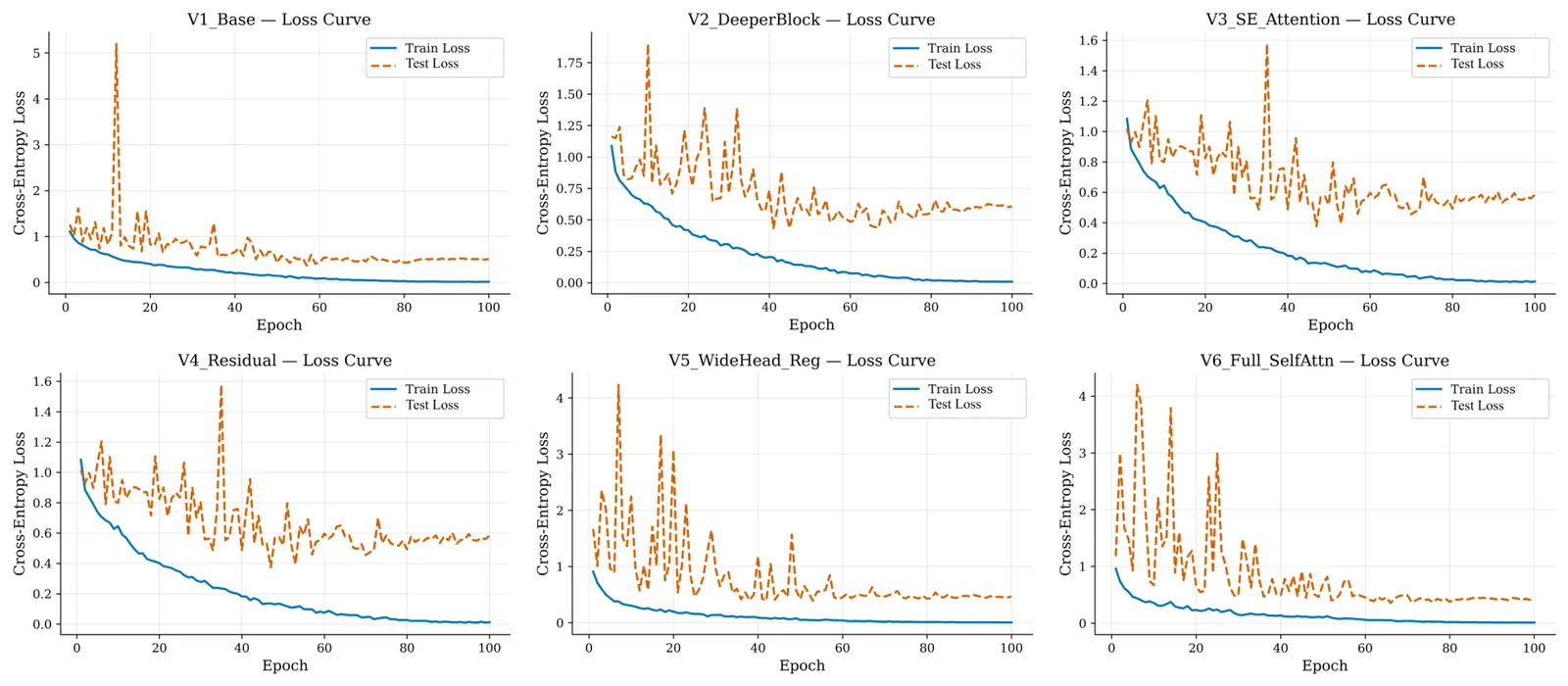
Trends in loss during training for different ablated variants.

**Figure 9:**
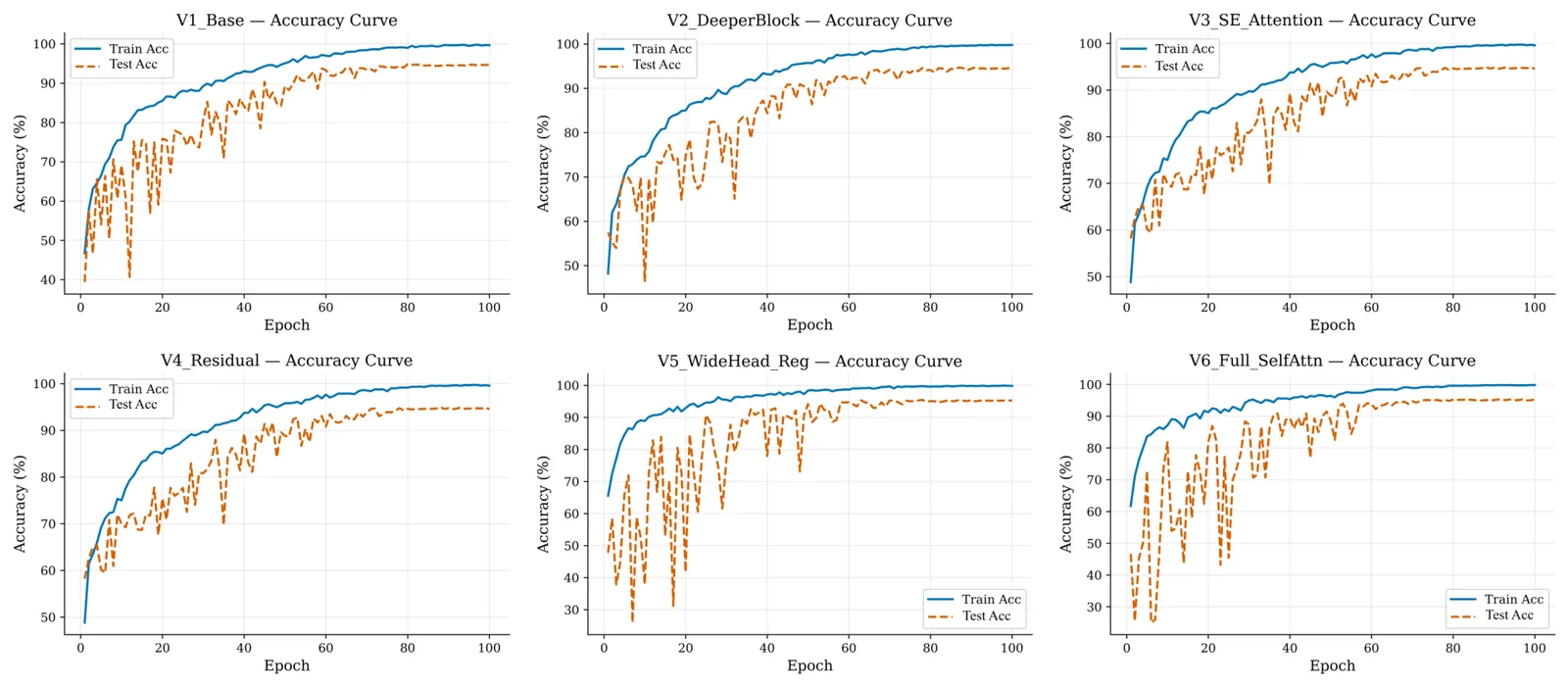
Trends in accuracy during training for different ablated variants.

**Figure 10:**
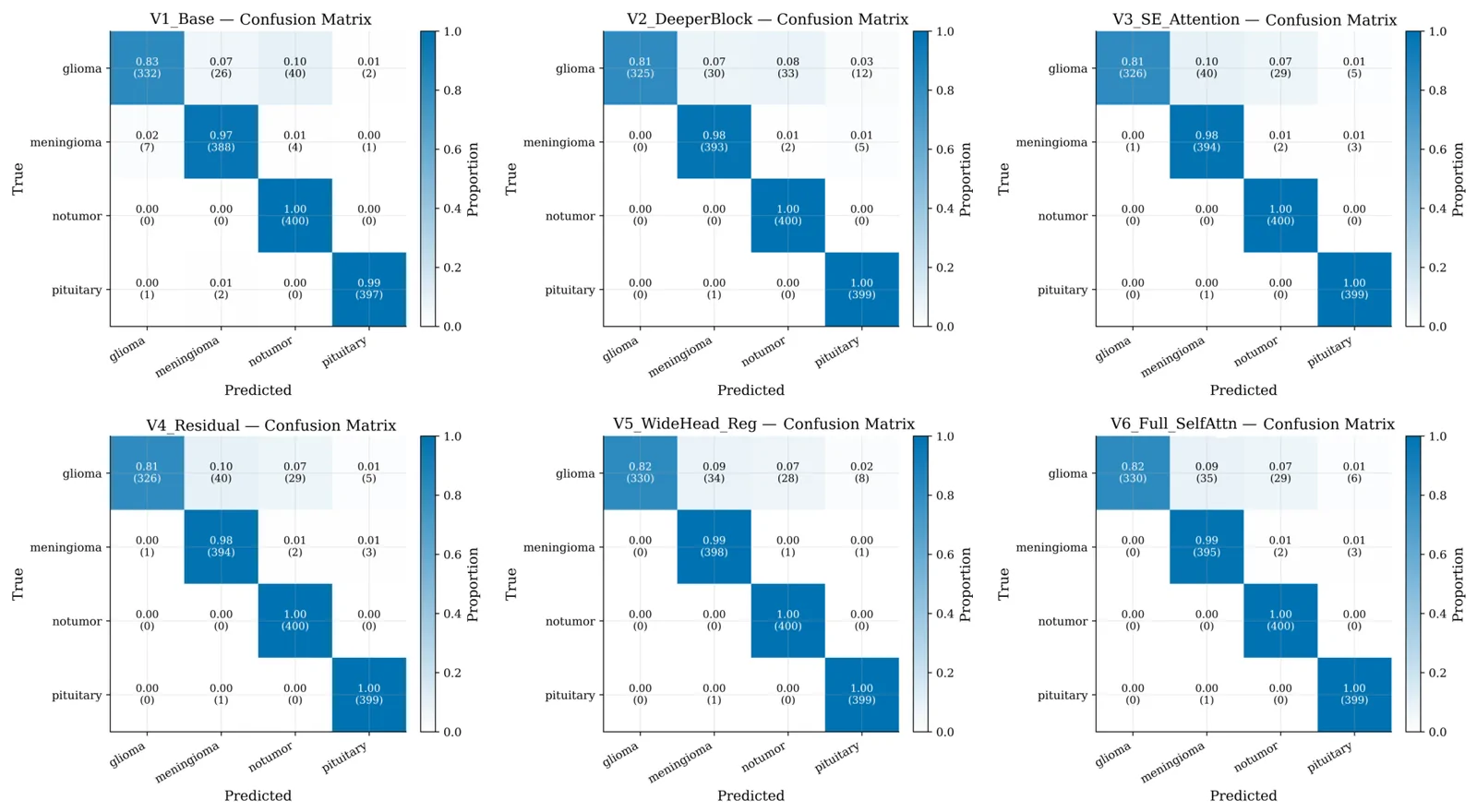
Normalized confusion matrices for different ablated variants.

Figure 8 and Figure 9 present the trends in loss and accuracy, respectively, for each variant. Figure 10 presents the confusion matrices for each variant.

Table 4 presents the overall classification performance of the ablated variants. Table 5 presents the per-class metrics for each variant. Table 6 presents the computational cost of the model. The baseline V1 achieved a test accuracy of 94.81%, along with an F1-score of 0.947. The integration of the fourth block increases the parameter count from 1.23 M to 4.94 M. However, the accuracy remains the same. Although AUC-ROC and PR-AUC were observed to improve slightly, various other metrics remained nearly the same. This suggests that the extra depth mainly helps the model separate the classes at the ranking level before it shows up as a hard-label accuracy gain. V3 adds SE attention, which results in a minimal increase in parameter count (i.e., 4.94 M→4.98 M). Accuracy and MCC, along with various other metrics, were observed to increase.

These results support the hypothesis that re-weighting helps the network focus on the subset of channels that carry tumor-related features, without incorporating additional computational cost. Integration of residual connection in V4 results in the same metrics as V3. This is an expected outcome rather than a bug. Residual connections mainly improve optimization by improving gradient flow rather than increasing the model’s representational capacity. Since the model contains only four blocks, vanishing gradient is not a major issue yet.

As a result, the benefits of skip connections become more visible only after the classification head is widened. Widening of the classification head from a single 128→64 layer to a two-layer 128→256→64 head, with BN and two dropout layers, produces the best classification results among all ablated variants. It achieved a test accuracy and F1-score of 95.44% and 0.953, respectively.

It achieved the highest precision, sensitivity, etc., along with the lowest FPR, FNR, etc., as shown in Table 7 and Table 8.

This analysis supports another underlying hypothesis of the model design. The deeper backbone with SE attention generates a richer 512-channel feature vector.

On the other hand, a single narrow classification layer cannot fully utilize this additional representational capacity. A wider head is able to utilize these features extensively. However, it must be properly regularized to prevent overfitting due to the increased number of parameters. Although V6 integrates a spatial self-attention model on top of V5, it does not provide a consistent performance. It decreases performance compared to V5. Spatial self-attention is most useful for capturing long-range dependencies in large or complex scenes. However, tumor regions are usually compact and are already represented by the convolutional layers. This representation is further improved by the SE blocks and residual connections included in V5. As a result, adding spatial self-attention increases model complexity without improving performance. This hypothesis is supported by the increased number of parameters (5.35 M vs. 5.02 M), higher inference time (2.81 ms vs. 2.78 ms per image), reduced throughput (343 img/s vs. 348 img/s), etc. In addition, the additional attention layers make optimization difficult, which leads to a higher number of epochs (91 vs. 78) and increased training time (88.10 min vs. 86.61 min) to reach convergence than V5.

**Figure 11:**
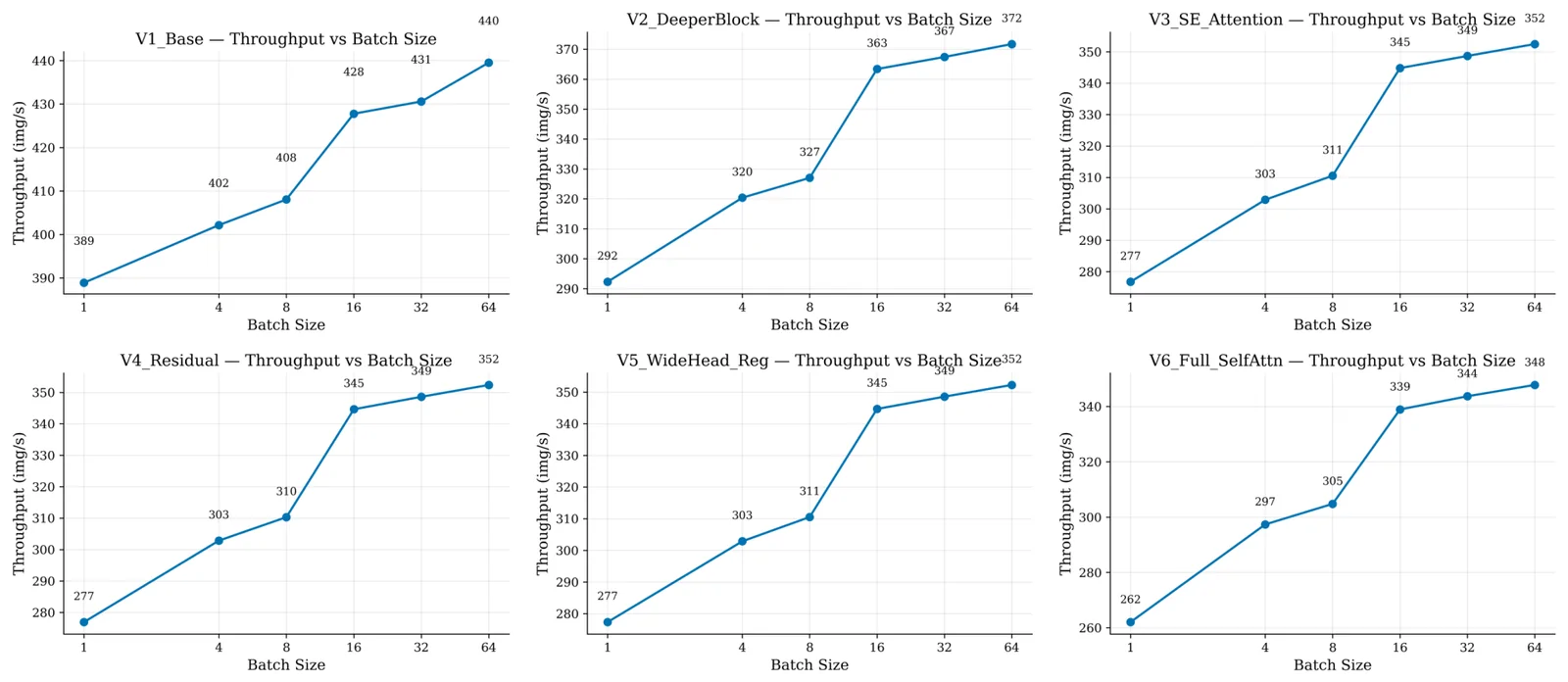
Trends in throughput against different batch sizes for different ablated variants.

Figure 11 visualizes the trends in throughput with change in batch size. A uniform growth was observed for all the variants, especially V3–V6, which reflects a very similar throughput curve at large batch sizes. This is because the throughput is dependent upon the similar four-block architecture rather than differences in the head or attention layers.

Table 9 provides a calibration view on the top of the classification metrics. V6 can be observed to achieve slightly better calibration metrics than V5 in terms of Informedness (0.937 vs. 0.939), Markedness (0.941 vs. 0.943), Threat Score (0.909 vs. 0.912), etc. As these improvements were very small, they do not directly translate into better classification performance.

In addition, they are not substantial enough to overlook the increased computational cost and reduced classification performance in V6. V5 achieved the lowest Brier score of all six variants. This indicates the trustworthiness of the predicted probabilities rather than relying on hard labels. These analyses indicate the effectiveness of V5 in terms of classification, calibration quality, computational efficiency, etc., for detecting BTs using MRI scans.

### 4.4. Comparison Against Pretrained Models

The efficacy of the proposed approach is evaluated against six widely used PTMs in order to quantify the competitiveness against established transfer-learning approaches. This comparison highlights whether the proposed architecture can achieve comparable or superior performance without depending upon pretraining on large datasets. This validates the effectiveness and practical applicability of the model against standard PTMs. The compared models include VGG16, ResNet50, DenseNet121, MobileNetV2, EfficientNetB0, and ShuffleNetV2. These models are particularly selected as they represent different generations, design philosophies, computational complexities, etc. This provides a comprehensive benchmark rather than a comparison against models with similar characteristics/family. Similar training mechanisms and environmental setups were used for all the models for a fair evaluation. A random seed of 42 was used during evaluation.

**Figure 12:**
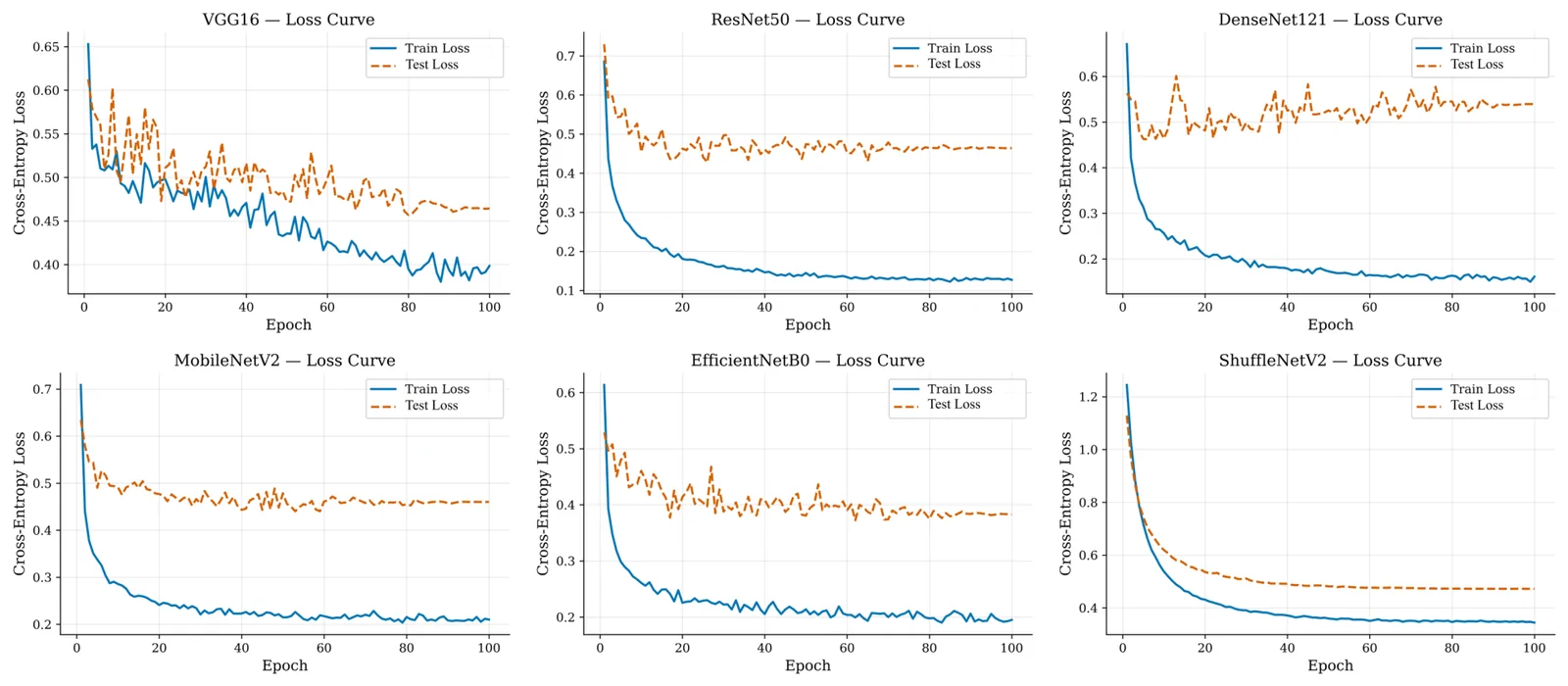
Trends in loss during training for different models.

**Figure 13:**
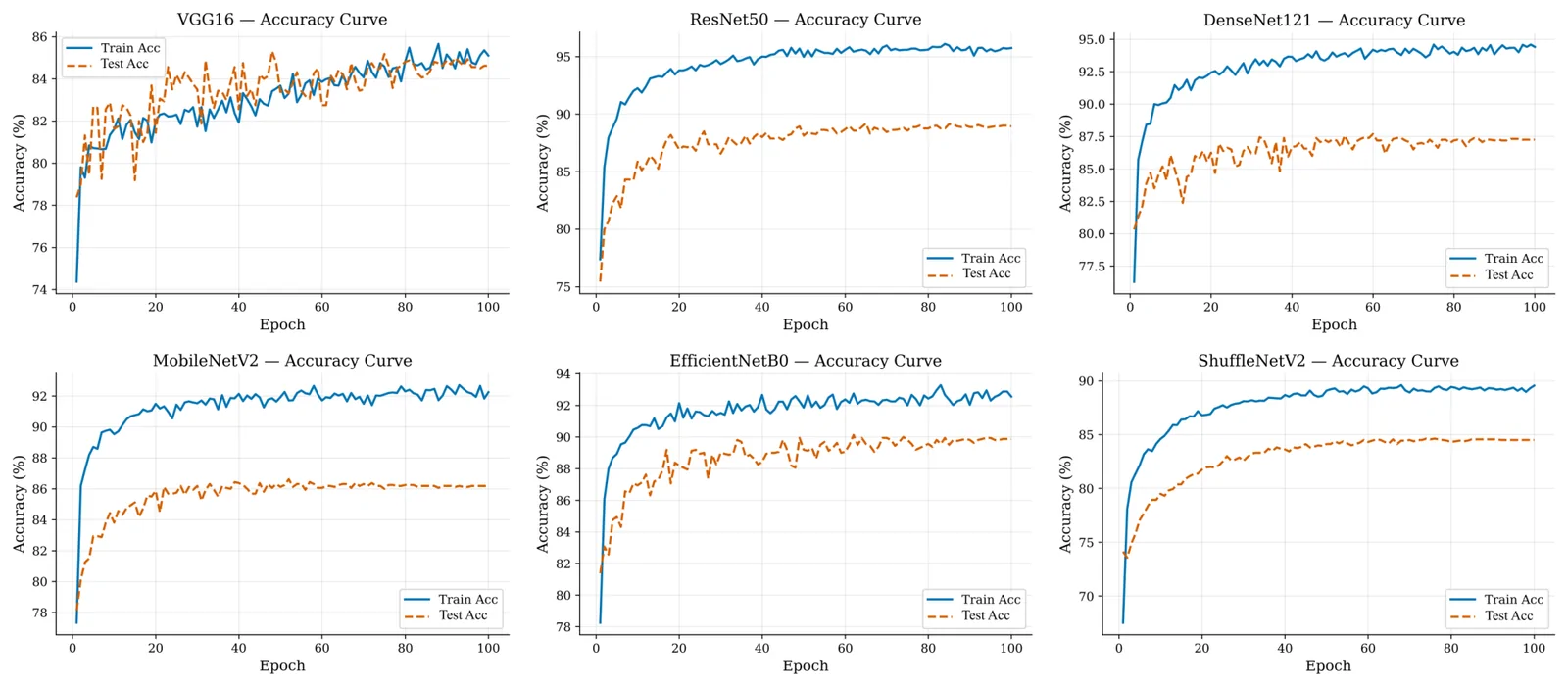
Trends in accuracy during training for different models.

**Figure 14:**
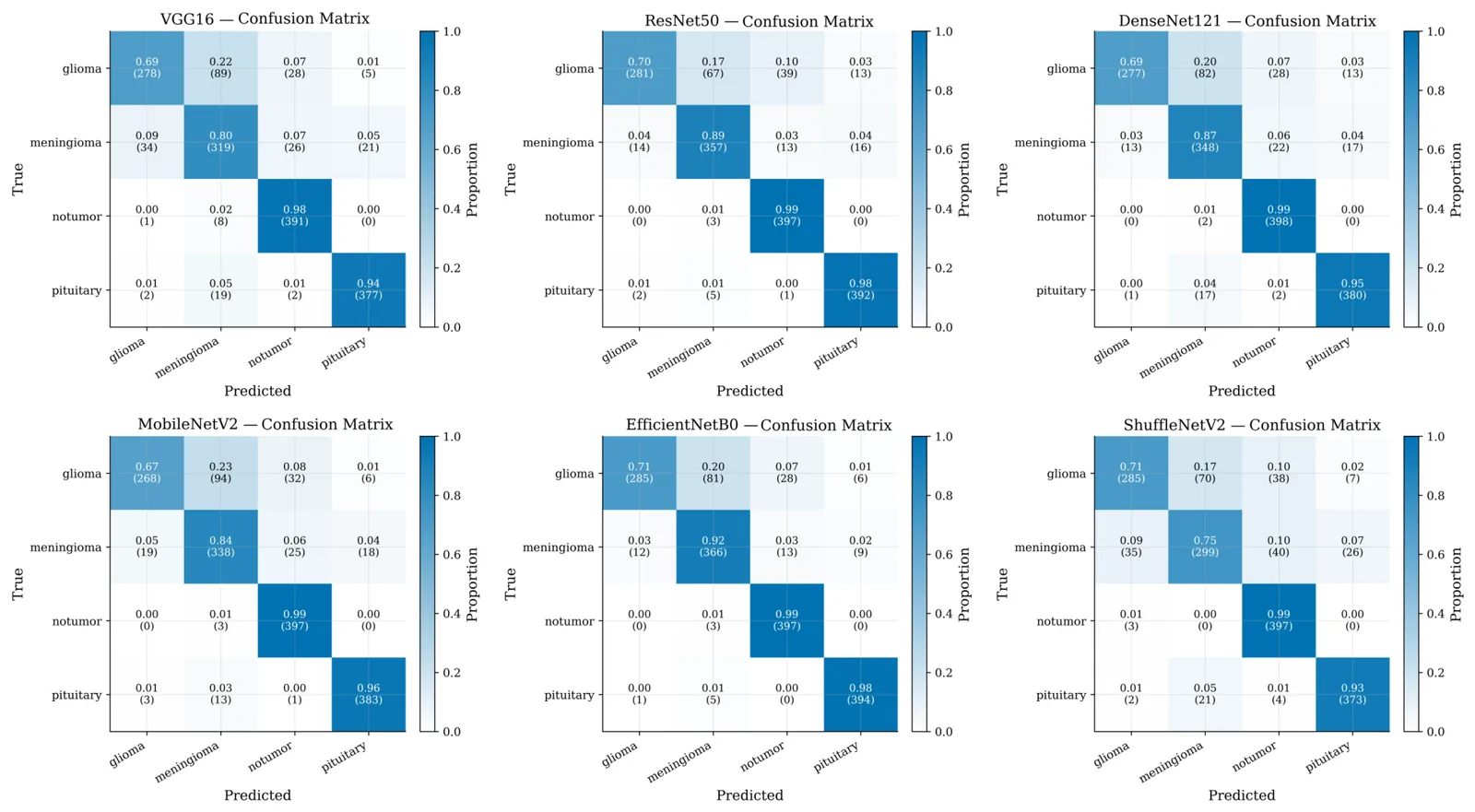
Normalized confusion matrices for different models.

Figure 12 and Figure 13 present the trends in loss and accuracy, respectively, during training. Figure 14 presents the confusion matrix of all the models.

Table 10 presents the overall classification metrics. The proposed model achieved an accuracy of 95.44%, compared to the best-performing PTM EfficientNetB0 with 90.13%. A similar statement could be made for the various other metrics, including AUC-ROC, PR-AUC, F1, and Cohen’s kappa. In addition, the trends in loss and accuracy indicate that the simulated PTMs memorize the training data and are ineffective for classification. Better training metrics but poor test metrics, along with a high gap between them, indicate potential overfitting.

Table 11 and Table 12 present additional classification metrics in order to quantify the performance against each class.

Even on Glioma (i.e., the hardest class for every model in comparison), the proposed model reaches an F1-score of 0.904 against 0.817. Similar results can be observed from Table 13 in terms of error analysis. The FPR and FNR of the proposed model are nearly a third of the corresponding rate for the best baseline, EfficientNetB0 (FPR 3.29%, FNR 9.88%), and less than a third of the worst baseline, ShuffleNetV2 (FPR 5.13%, FNR 15.38%). Table 14 presents the diagnostic metrics of the simulated models.

The computational metrics in Table 15 and Table 16 highlight a major gap among all models.

**Figure 15:**
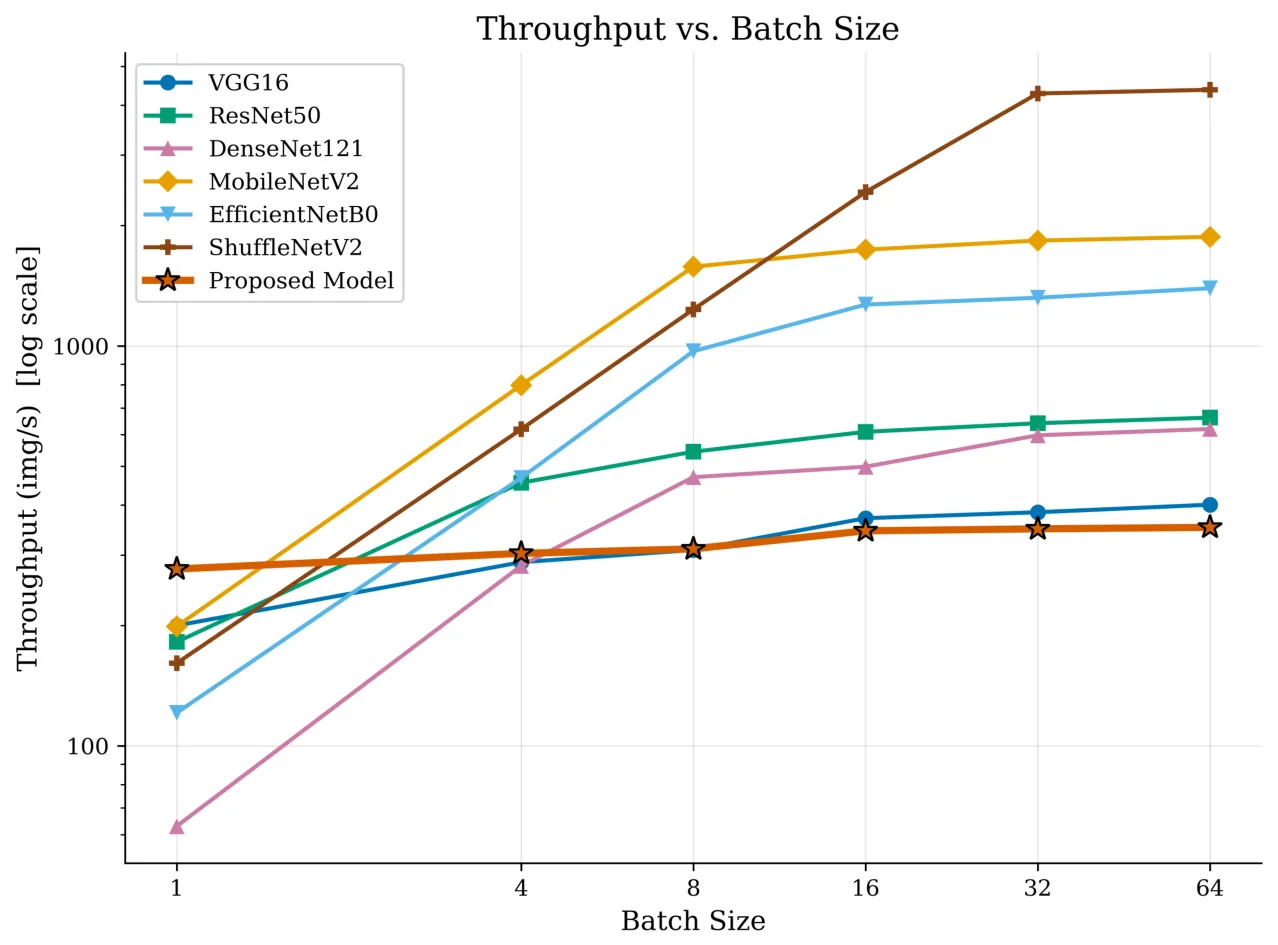
Trends in throughput under different batch sizes for different models.

Figure 15 presents the trends in throughput for different PTMs on varying batch sizes. For every PTM, the number of training parameters is a tiny portion of the overall parameter count, with a major chunk occupied by non-trainable parameters. It is a result of pre-learned features from the ImageNet dataset that enable their diversification.

ImageNet consists of natural photographs like food, real-world objects, vehicles, etc. As a result, the frozen background does not contribute much to adaptation for a medical grayscale image with a limited Region of Interest (ROI) to enable decision. This highlights that the classification decision for this specific task (BTC) relies on this small portion. Since most parameters are frozen, the model cannot sufficiently adjust its learned features to the new dataset. On the other hand, utilizing all the parameters as trainable enables the model to focus solely on the task. This breakdown of parameters is presented in Table 15. The trainable share for every PTM baseline is a small fraction of its total parameter count. For example, ResNet50 has only 8,196 trainable parameters out of 23.5 million. On the other hand, the proposed model keeps all of its ≈5 million parameters trainable. It allows every layer to adapt directly to the tumor-classification task instead of depending on features learned from an unrelated image domain. The proposed model achieved ≈96% fewer parameters, compared to VGG16 (i.e., the one with the lowest training epochs) and ≈25% fewer parameters than EfficientNetB0 (i.e., the one with the best classification metrics). On the other hand, the proposed model needs 21.8 GFLOPs per image. Although this is more than ResNet50 and various other models, it is below VGG16 (30.9). This distinction matters because parameter count and computational cost do not always move together. A model can hold few parameters yet still require many FLOPs per image if most of its layers stay fully trainable and process high-resolution feature maps. The proposed approach makes a trade-off here. Despite the increase in computation metrics, the proposed approach achieves a balanced workflow over high-throughput achievers like ShuffleNetV2. It is considered as a suitable choice, especially in terms of parameter-efficiency. Although models like MobileNetV2 and ShuffleNetV2 remain considerably cheaper to run despite having a comparable or higher parameter count, their slightly poor classification performance inhibits their deployment. This parameter efficiency does not directly translate into the lowest computational cost, since the proposed model performs all convolutions on trainable weights rather than delegating most of the computation to frozen layers. Still, a near-uniform throughput across different batches, even in small ones, indicates the reasonable trade-off in terms of computational cost and classification performance.

### 4.5. Reproducibility Statistical Analysis

All the previous tests were evaluated under a single random seed. In order to evaluate the stability of the model under different internal variations, a reproducibility analysis was conducted using different random seeds. This test ensures that the results are not a byproduct of lucky random initializations or data splits. This also ensures that the results are not biased towards a single seed. The analysis was conducted on four different seeds: 0, 42, 123, and 2026. It included widely used seeds like 0 and 42, along with arbitrary seeds like 123 and 2026. These seeds span different numerical values and digit lengths for a diverse evaluation. The model was re-executed for seed 42 to evaluate reproducibility. All the tests were independent and un-influenced by other runs. Similar training mechanisms and environmental setups were used for all the models for a fair evaluation.

Table 17 presents per-class metrics of the proposed model in terms of mean ± standard deviation. Accuracy per class stays on the top with the lowest standard deviation of less than 0.5% for every class. The same could be said for various other metrics as well. Pituitary was observed to be seed-independent with sensitivity 0.997±0.003 and specificity 0.992±0.001. Its distinct anatomical location provides the model a strong and consistent signal to learn, regardless of weight. On the other hand, the sensitivity of Glioma reported the highest sensitivity at 0.832±0.017. However, this is consistent with the earlier-discussed observation that Glioma is the hardest class to recall and is naturally more sensitive to exactly which decision boundary a given random initialization converges to. Brier loss indicates the same hierarchy. Despite this, a low standard deviation across multiple metrics indicates the stability of the proposed approach while making classification decisions.

### 4.6. Robustness to Test-Time Perturbations

After deploying a model, there is no guarantee that the input image is always clean and well formatted. Since the model is trained and evaluated on clean images, the model may fall short against perturbations unless well augmented during the training period. In order to quantify the strength of the model in such scenarios, an analysis against fifteen commonly used image perturbations was conducted. Table 18 presents the details of the simulated perturbations.

These perturbations and their intensity were chosen to simulate realistic variations encountered during image acquisition and deployment. It was ensured that the degradations were perceptible yet did not make the images unrecognizable. For a fair evaluation, images from the test set (no images from the train set were involved) were selected for evaluation. Table 19 presents the prediction analysis on four randomly selected images, one per class, where ✔ and × indicates correct and incorrect classification, respectively. Table 20 presents the average classification metrics for the entire test set.

The sample images passed almost all perturbations with flying colors, except the ones for Glioma. The low performance of the Glioma sample aligns with the earlier discussed limitation. These perturbations made the decision for it even more difficult than the clean ones. One observable factor in these analyses is that the color-space perturbations do not affect the classification, i.e., an accuracy of 95.44%. This is an expected result, as MRI scans are grayscale in nature. As a result, the model that relies on structural and texture patterns rather than color information should be unaffected. This is what the proposed model interprets. On the other hand, geometric perturbations affect the model in different terms. Horizontal flip causes negligible degradation, with a drop in accuracy of 0.19%. Vertical flip was found to be the most damaging perturbation tested that resulted in an accuracy drop of 13.81%. As the training augmentation pipeline includes horizontal flipping but not vertical flipping, the network is trained on left–right mirroring but not on upside-down brain scans. As a result, this drop does not represent a gap in classification performance but highlights the need for wider training augmentations. This hypothesis is also supported by the results of rotation. A rotation of $15^{\circ }$ was used in the training augmentation, but evaluation on $20^{\circ }$ results in a minimal loss of 1%. This shows consistency with the generalization capability of the model within/near the range of perturbations it was trained on. Noise-based perturbations, including Gaussian blur cause a 10.56% drop in accuracy, i.e., the second-most-disturbing perturbation. Since the boundaries of the tumor are defined by fine edge and texture details, blurring completely destroys them. This highlights the need for a more extensive and diverse range of augmentations in order to make the approach robust against different image perturbations.

### 4.7. Considerations and Constraints

The above-discussed analyses of various experiments highlight the efficacy of the approach for classification of BTs. The exact-duplicate check shows that no images were duplicates in the dataset. In addition, various existing studies also highlight the presence of no duplicate images in this dataset [25]. However, this analysis cannot detect two different files that show the same underlying slice, i.e., the same scan re-exported at a different resolution or compression level. Since the merge dataset does not provide the patient identifiers, splitting the data as per patients was not possible. As an additional level of check, a perceptual similarity check was carried out between the training and test images. Each image was reduced to a 64-bit average-hash and a 64-bit difference-hash. A test image was flagged whenever its hash fell within a Hamming distance of five bits from any training image of the same class. Table 21 summarizes the outcome of this experiment. A sizable portion of test images were flagged under this scenario. However, this cannot be interpreted entirely as the presence of duplicate images. Brain MRI slices from the same class share a broadly similar skull outline, tumor-free brain structure, and acquisition geometry even when they come from different patients. As a result, a purely appearance-based hash is expected to consider many same-class images to be similar regardless of any actual overlap. Removing them directly with the aim of improved training can result in removal of actual MRI images as well [26]. This study acknowledges this limitation. In order to remove it without the presence of patient-aware metadata, similarity-based data leakage screening can be incorporated. Collectively, SHA-256, perceptual hashing, Structural Similarity Index (SSIM), and CNN embeddings, followed by their clustering, can be utilized in future studies. The availability of patient-level data in MRI cohorts can help improve model training and evaluate the effectiveness of the similarity-based data-leakage screening approach before practical deployment. In addition, the proposed model is trained and evaluated on a single publicly available dataset. The geographical distribution and demographic details of the patients represented in the selected dataset are not clearly reported. This makes it difficult to assess potential geographic or population-related biases. As a result, the robustness of the proposed SERA-Net for diverse patient populations and healthcare settings cannot be fully assessed. Further validation on a diverse, multi-geographic dataset with well-documented characteristics would be beneficial to establish the broader applicability of the proposed approach.

## 5. Conclusions and Future Work

This work presents a convolutional architecture, built from scratch, for BTC using MRI scans. Its individual components, i.e., Squeeze-and-Excite attention, residual connections, and a wider classifier head, are all established techniques; the contribution lies in combining them through a controlled, incremental ablation process and in highlighting the contribution of each one. It was trained and evaluated on a publicly available brain MRI dataset. It achieved a test accuracy of 95.44%, MCC of 0.940, along with near-perfect per-class analysis, especially for the pituitary and no-tumor classes. An analysis against six PTMs trained under similar environments showed a consistent and visible advantage over them. It required ≈96% and ≈25% less parameters than VGG16 and EfficientNetB0, respectively, while achieving a relative improvement in accuracy of ≈10% and ≈5%, respectively. This parameter efficiency comes with a cost of higher per-image FLOPs, i.e., 21.8 GFLOPs per image, compared to compact mobile-oriented architectures (0.61 GFLOPs and 0.29 GFLOPs for MobileNetV2 and ShuffleNetV2, respectively). This is mainly because the model processes high-resolution feature maps and keeps all layers trainable. This trade-off should be considered when the target deployment environments are more affected by inference latency rather than model size. Since the model is trained on a single dataset, the true efficiency of the model needs to be measured against multiple datasets. Performance evaluation on real-world imaging devices, supported by an extensive augmentation strategy, is a fruitful research direction. In addition, making the approach lighter while preserving the performance for resource-constrained devices is another fruitful working direction.

## Figures and Tables

**Table 1:** Layer-wise input/output shapes and parameter counts of the proposed model.

Block	Layer	Input Shape	Output Shape	Parameters
Block 1 (3 → 64)	Conv2d (3 × 3, 3 → 64)	224×224×3	224×224×64	1792
	BatchNorm2d (64)	224×224×64	224×224×64	128
	Conv2d (3 × 3, 64 → 64)	224×224×64	224×224×64	36,928
	BatchNorm2d (64)	224×224×64	224×224×64	128
	SE Block (64, r = 16)	224×224×64	224×224×64	1096
	Projection Conv (1 × 1, 3 → 64)	224×224×3	224×224×64	256
	MaxPool2d (2 × 2)	224×224×64	112×112×64	0
Block 2 (64 → 128)	Conv2d (3 × 3, 64 → 128)	112×112×64	112×112×128	73,856
	BatchNorm2d (128)	112×112×128	112×112×128	256
	Conv2d (3 × 3, 128 → 128)	112×112×128	112×112×128	147,584
	BatchNorm2d (128)	112×112×128	112×112×128	256
	SE Block (128, r = 16)	112×112×128	112×112×128	2184
	Projection Conv (1 × 1, 64 → 128)	112×112×64	112×112×128	8320
	MaxPool2d (2 × 2)	112×112×128	56×56×128	0
Block 3 (128 → 256)	Conv2d (3 × 3, 128 → 256)	56×56×128	56×56×256	295,168
	BatchNorm2d (256)	56×56×256	56×56×256	512
	Conv2d (3 × 3, 256 → 256)	56×56×256	56×56×256	590,080
	BatchNorm2d (256)	56×56×256	56×56×256	512
	SE Block (256, r = 16)	56×56×256	56×56×256	8464
	Projection Conv (1 × 1, 128 → 256)	56×56×128	56×56×256	33,024
	MaxPool2d (2 × 2)	56×56×256	28×28×256	0
Block 4 (256 → 512)	Conv2d (3 × 3, 256 → 512)	28×28×256	28×28×512	1,180,160
	BatchNorm2d (512)	28×28×512	28×28×512	1024
	Conv2d (3 × 3, 512 → 512)	28×28×512	28×28×512	2,359,808
	BatchNorm2d (512)	28×28×512	28×28×512	1024
	SE Block (512, r = 16)	28×28×512	28×28×512	33,312
	Projection Conv (1 × 1, 256 → 512)	28×28×256	28×28×512	131,584
	MaxPool2d (2 × 2)	28×28×512	14×14×512	0
Feature Embedding	GAP	14×14×512	1×1×512	0
	Linear (512 → 128) + ReLU	512	128	65,664
Classification Head	Linear (128 → 256)	128	256	33,024
	BatchNorm1d (256)	256	256	512
	Linear (256 → 64)	256	64	16,448
	BatchNorm1d (64)	64	64	128
	Linear (64 → 4)	64	4	260
Total Parameters				5,023,492

**Table 2:** Per-class classification metrics for the proposed model.

Class	Precision	Recall	F1-Score
Glioma	1.000	0.825	0.904
Meningioma	0.919	0.995	0.956
No Tumor	0.932	1.000	0.965
Pituitary	0.978	0.998	0.988
Macro Avg	0.957	0.954	0.953

**Table 3:** Details of six simulated ablated variants.

Variant	Component Added	Role in the Evaluation
V1	Baseline: 3 convolutional blocks (64–128–256 channels) + GAP + simple head (one 128 → 64)	Used to establish the lower bound of performance and to isolate the effect of each later addition.
V2	4th convolutional block (256 → 512 channels)	Measures whether increased network depth alone improves discrimination between visually similar tumor classes.
V3	SE attention in every convolutional block	Measures the contribution of channel-wise attention to feature selectivity, independent of depth or residual connections.
V4	Residual (skip) connections inside each convolutional block	Measures whether residual connections improve training stability and accuracy on top of the deeper, attention-equipped backbone.
V5	Wider classifier head (128 → 256 → 64 → C) with BN and Dropout (0.5, 0.3)	Measures the effect of head capacity and stronger regularization once the backbone improvements are already in place.
V6	Spatial self-attention block before GAP	Measures whether spatial (non-local) attention adds further benefit on top of all previous improvements.

**Table 4:** Overall classification performance of the different ablated variants.

Variant	Accuracy (%)	F1	MCC	AUC-ROC	PR-AUC	BalancedAcc	CohenKappa
V1	94.813	0.947	0.932	0.987	0.976	0.965	0.930
V2	94.813	0.947	0.932	0.988	0.978	0.965	0.930
V3	94.938	0.948	0.934	0.987	0.975	0.966	0.931
V4	94.938	0.948	0.934	0.987	0.975	0.966	0.931
V5	95.438	0.953	0.940	0.986	0.980	0.970	0.938
V6	95.250	0.951	0.938	0.986	0.980	0.968	0.936

**Table 5:** Per-class performance metrics for different ablated variants.

Class	Metric	V1	V2	V3	V4	V5	V6
Glioma	Precision	0.976	1.000	0.997	0.997	1.000	1.000
	Recall	0.830	0.812	0.815	0.815	0.825	0.825
	F1-score	0.897	0.897	0.897	0.897	0.904	0.904
Meningioma	Precision	0.933	0.927	0.906	0.906	0.919	0.916
	Recall	0.970	0.983	0.985	0.985	0.995	0.988
	F1-score	0.951	0.954	0.944	0.944	0.956	0.951
No Tumor	Precision	0.901	0.920	0.928	0.928	0.932	0.928
	Recall	1.000	1.000	1.000	1.000	1.000	1.000
	F1-score	0.948	0.958	0.963	0.963	0.965	0.963
Pituitary	Precision	0.993	0.959	0.980	0.980	0.978	0.978
	Recall	0.993	0.998	0.998	0.998	0.998	0.998
	F1-score	0.993	0.978	0.989	0.989	0.988	0.988
Macro Avg	Precision	0.951	0.951	0.953	0.953	0.957	0.956
	Recall	0.948	0.948	0.949	0.949	0.954	0.953
	F1-score	0.947	0.947	0.948	0.948	0.953	0.951
Weighted Avg	Precision	0.951	0.951	0.953	0.953	0.957	0.956
	Recall	0.948	0.948	0.949	0.949	0.954	0.953
	F1-score	0.947	0.947	0.948	0.948	0.953	0.951

**Table 6:** Computational complexity and efficiency comparison of different ablated variants.

Variant	Params Total (M)	Params Trainable (M)	Params Non-Trainable (M)	Train Time (min)	Inf_ms	Throughput (img/s)	Best Epoch
V1	1.230	1.230	0	70.269	2.235	430.591	80
V2	4.937	4.937	0	80.678	2.641	367.422	100
V3	4.982	4.982	0	86.620	2.783	348.663	88
V4	4.982	4.982	0	86.581	2.783	348.601	88
V5	5.023	5.023	0	86.618	2.775	348.564	78
V6	5.352	5.352	0	88.104	2.813	343.679	91

**Table 7:** Discrimination metrics for each ablated variant.

Variant	Precision (PPV)	Sensitivity (TPR)	Specificity (TNR)	NPV	F2	F0.5
V1	0.951	0.948	0.983	0.983	0.947	0.949
V2	0.951	0.948	0.983	0.984	0.947	0.949
V3	0.953	0.949	0.983	0.984	0.948	0.950
V4	0.953	0.949	0.983	0.984	0.948	0.950
V5	0.957	0.954	0.985	0.986	0.953	0.955
V6	0.956	0.953	0.984	0.985	0.951	0.953

**Table 8:** Error rate analysis of different ablated variants.

Variant	FPR	FNR	FDR	FOR
V1	0.017	0.052	0.049	0.017
V2	0.017	0.052	0.049	0.016
V3	0.017	0.051	0.047	0.016
V4	0.017	0.051	0.047	0.016
V5	0.015	0.046	0.043	0.014
V6	0.016	0.048	0.044	0.015

**Table 9:** Additional diagnostic and calibration metrics of different ablated variants.

Variant	Informedness (J)	Markedness	ThreatScore (CSI)	PLR	NLR	DOR	BrierScore	Log Loss
V1	0.931	0.934	0.902	147.586	0.052	inf	0.023	0.408
V2	0.931	0.935	0.900	inf	0.052	inf	0.024	0.531
V3	0.933	0.937	0.903	298.791	0.051	inf	0.024	0.506
V4	0.933	0.937	0.903	298.791	0.051	inf	0.024	0.506
V5	0.939	0.943	0.912	inf	0.046	inf	0.021	0.426
V6	0.937	0.941	0.909	inf	0.048	inf	0.022	0.409

**Table 10:** Overall classification performance: proposed model vs. pretrained baselines.

Model	Accuracy (%)	F1	MCC	AUC-ROC	PR-AUC	BalancedAcc	CohenKappa
VGG16	85.313	0.851	0.806	0.957	0.907	0.902	0.803
ResNet50	89.188	0.888	0.858	0.974	0.948	0.928	0.853
DenseNet121	87.688	0.874	0.839	0.969	0.938	0.918	0.834
MobileNetV2	86.625	0.863	0.824	0.968	0.928	0.911	0.819
EfficientNetB0	90.125	0.899	0.871	0.977	0.944	0.934	0.866
ShuffleNetV2	84.625	0.843	0.796	0.960	0.911	0.898	0.792
Proposed Model	95.438	0.953	0.940	0.986	0.980	0.970	0.938

**Table 11:** Discrimination metrics of the simulated models.

Model	Precision (PPV)	Sensitivity (TPR)	Specificity (TNR)	NPV	F2	F0.5
VGG16	0.857	0.853	0.951	0.952	0.851	0.853
ResNet50	0.896	0.892	0.964	0.966	0.889	0.892
DenseNet121	0.885	0.877	0.959	0.961	0.874	0.878
MobileNetV2	0.873	0.866	0.955	0.957	0.864	0.867
EfficientNetB0	0.908	0.901	0.967	0.969	0.899	0.902
ShuffleNetV2	0.848	0.846	0.949	0.950	0.844	0.845
Proposed Model	0.957	0.954	0.985	0.986	0.953	0.955

**Table 12:** Per-class classification metrics of the simulated models.

Class	Metric	VGG16	ResNet50	DenseNet121	MobileNetV2	EfficientNetB0	ShuffleNetV2	Proposed Model
Glioma	Precision	0.883	0.946	0.952	0.924	0.956	0.877	1.000
	Recall	0.695	0.703	0.693	0.670	0.713	0.713	0.825
	F1-score	0.778	0.806	0.802	0.777	0.817	0.786	0.904
Meningioma	Precision	0.733	0.826	0.775	0.754	0.804	0.767	0.919
	Recall	0.797	0.892	0.870	0.845	0.915	0.748	0.995
	F1-score	0.764	0.858	0.820	0.797	0.856	0.757	0.956
No Tumor	Precision	0.875	0.882	0.884	0.873	0.906	0.829	0.932
	Recall	0.978	0.993	0.995	0.993	0.993	0.993	1.000
	F1-score	0.923	0.934	0.936	0.929	0.947	0.903	0.965
Pituitary	Precision	0.935	0.931	0.927	0.941	0.963	0.919	0.978
	Recall	0.943	0.980	0.950	0.958	0.985	0.932	0.998
	F1-score	0.939	0.955	0.938	0.949	0.974	0.926	0.988
Macro Avg	Precision	0.857	0.896	0.885	0.873	0.908	0.848	0.957
	Recall	0.853	0.892	0.877	0.866	0.901	0.846	0.954
	F1-score	0.851	0.888	0.874	0.863	0.899	0.843	0.953
Weighted Avg	Precision	0.857	0.896	0.885	0.873	0.908	0.848	0.957
	Recall	0.853	0.892	0.877	0.866	0.901	0.846	0.954
	F1-score	0.851	0.888	0.874	0.863	0.899	0.843	0.953

**Table 13:** Error rate analysis of different simulated models.

Model	FPR	FNR	FDR	FOR
VGG16	0.049	0.147	0.143	0.048
ResNet50	0.036	0.108	0.104	0.034
DenseNet121	0.041	0.123	0.115	0.039
MobileNetV2	0.045	0.134	0.127	0.043
EfficientNetB0	0.033	0.099	0.092	0.031
ShuffleNetV2	0.051	0.154	0.152	0.050
Proposed Model	0.015	0.046	0.043	0.014

**Table 14:** Additional diagnostic and calibration metrics of the different simulated models.

Model	Informedness (J)	Markedness	ThreatScore (CSI)	PLR	NLR	DOR	BrierScore	Log Loss
VGG16	0.804	0.809	0.749	23.809	0.155	434.016	0.057	0.480
ResNet50	0.856	0.862	0.804	32.498	0.111	1285.438	0.042	0.431
DenseNet121	0.836	0.845	0.782	32.664	0.127	1349.477	0.047	0.511
MobileNetV2	0.822	0.830	0.767	28.543	0.140	968.068	0.051	0.450
EfficientNetB0	0.868	0.876	0.822	46.489	0.101	2322.291	0.039	0.373
ShuffleNetV2	0.795	0.798	0.735	19.916	0.162	600.185	0.061	0.474
Proposed Model	0.939	0.943	0.912	inf	0.046	inf	0.021	0.426

**Table 15:** Parameter count, trainable parameters, and inference efficiency: proposed model vs. pretrained baselines.

Model	ParamsTotal (M)	Params Trainable (M)	Params NonTrainable (M)	Train Time (min)	Inf ms	Throughput (img/s)	Best Epoch
VGG16	134.277	0.016	134.261	38.630	2.577	384.281	48
ResNet50	23.516	0.008	23.508	34.041	1.565	640.720	65
DenseNet121	6.958	0.004	6.954	35.656	1.963	598.301	60
MobileNetV2	2.229	0.005	2.224	32.414	0.566	1835.276	52
EfficientNetB0	4.013	0.005	4.008	33.150	0.780	1320.406	62
ShuffleNetV2	1.258	0.004	1.254	32.389	0.429	4278.048	74
Proposed Model	5.023	5.023	0	86.618	2.775	348.564	78

**Table 16:** Computational complexity of the various simulated models.

Variant	Params (M)	Size (MB)fp32	Size (MB)fp16	GMACs	GFLOPs	FLOPs	MACs	FLOPs/ Param	Params/ GFLOP (M)
VGG16	134.277	537.108	268.554	15.466	30.932	30,932,369,408	15,466,184,704	230.363	4.341
ResNet50	23.516	94.065	47.032	4.098	8.197	8,196,516,864	4,098,258,432	348.547	2.869
DenseNet121	6.958	27.832	13.916	2.849	5.698	5,697,618,432	2,848,809,216	818.864	1.221
MobileNetV2	2.229	8.916	4.458	0.306	0.612	612,355,008	306,177,504	274.722	3.640
EfficientNetB0	4.013	16.051	8.025	0.391	0.783	782,555,136	391,277,568	195.021	5.128
ShuffleNetV2	1.258	5.031	2.515	0.146	0.292	291,676,576	145,838,288	231.912	4.312
Proposed Model	5.023	20.094	10.047	10.908	21.816	21,816,040,576	10,908,020,288	4342.804	0.230

**Table 17:** Reproducibility analysis of the proposed approach on four random seeds.

Group	Metric	Glioma	Meningioma	No Tumor	Pituitary
Overall Performance	Accuracy (%)	95.720 ± 0.320	97.780 ± 0.420	97.970 ± 0.400	99.340 ± 0.040
	Balanced Accuracy	0.915 ± 0.008	0.981 ± 0.002	0.987 ± 0.003	0.995 ± 0.001
Core Rates	Sensitivity (TPR)	0.832 ± 0.017	0.988 ± 0.008	1.000 ± 0.000	0.997 ± 0.003
	Specificity (TNR)	0.999 ± 0.002	0.975 ± 0.008	0.973 ± 0.005	0.992 ± 0.001
	Precision (PPV)	0.996 ± 0.006	0.929 ± 0.021	0.925 ± 0.014	0.977 ± 0.004
	NPV	0.947 ± 0.005	0.996 ± 0.003	1.000 ± 0.000	0.999 ± 0.001
Error Rates	FPR	0.001 ± 0.002	0.025 ± 0.008	0.027 ± 0.005	0.008 ± 0.001
	FNR	0.168 ± 0.017	0.013 ± 0.008	0.000 ± 0.000	0.003 ± 0.003
	FDR	0.004 ± 0.006	0.071 ± 0.021	0.075 ± 0.014	0.023 ± 0.004
	FOR	0.053 ± 0.005	0.004 ± 0.003	0.000 ± 0.000	0.001 ± 0.001
Composite Scores	F1-Score	0.907 ± 0.008	0.957 ± 0.008	0.961 ± 0.007	0.987 ± 0.001
	F2-Score	0.860 ± 0.013	0.975 ± 0.003	0.984 ± 0.003	0.993 ± 0.002
	F0.5-Score	0.958 ± 0.002	0.940 ± 0.016	0.939 ± 0.011	0.981 ± 0.003
	Threat Score (CSI)	0.829 ± 0.013	0.918 ± 0.014	0.925 ± 0.014	0.974 ± 0.001
Agreement Metrics	MCC	0.885 ± 0.008	0.943 ± 0.010	0.949 ± 0.010	0.983 ± 0.001
	Cohen’s Kappa	0.879 ± 0.010	0.942 ± 0.011	0.947 ± 0.010	0.983 ± 0.001
	Informedness (J)	0.831 ± 0.015	0.962 ± 0.003	0.973 ± 0.005	0.989 ± 0.002
	Markedness	0.943 ± 0.003	0.925 ± 0.018	0.925 ± 0.014	0.976 ± 0.003
Ranking Metrics	AUC-ROC	0.952 ± 0.003	0.996 ± 0.001	0.998 ± 0.001	1.000 ± 0.000
	PR-AUC	0.938 ± 0.002	0.983 ± 0.008	0.992 ± 0.003	0.999 ± 0.001
Calibration	Brier Score	0.042 ± 0.003	0.020 ± 0.003	0.019 ± 0.004	0.005 ± 0.001

**Table 18:** Details of the test-time perturbations applied during the robustness evaluation.

Perturbation	Parameter Value (s)
No Attack	Clean, unmodified test image (baseline).
Horizontal Flip	Mirrored left-right.
Vertical Flip	Mirrored top-bottom.
Affine Transform	Shear coefficients (a,b,c,d,e,f)=(1.0,0.08,−0.03w,0.05,1.0,−0.03h), bilinear resampling.
Rotation	Rotated by $20^{\circ }$, bilinear resampling, fill color (0,0,0).
Gaussian Blur	Gaussian blur, radius =2.0 px.
Motion Blur	Horizontal motion-blur kernel, size k=9.
Speckle Noise	Multiplicative Gaussian noise, $N(0,0.15^{2})$.
Gaussian Noise	Additive Gaussian noise, $N(0,0.08^{2})$.
Contrast Shift	Contrast enhancement factor =0.5 (reduced contrast).
Saturation Shift	Color/saturation enhancement factor =0.3 (desaturated).
Brightness Shift	Brightness enhancement factor =1.5 (increased brightness).
Hue Shift	Hue channel (HSV) shifted by +60 (mod 256).
JPEG Compression	Re-encoded at JPEG quality =10.
Random Erasing	Random rectangular region of size 0.3h×0.3w set to 0 (black), random location.

**Table 19:** Prediction correctness under different test-time perturbations for sample images.

Image Name → True_Label →	Te-gl_1.jpg Glioma	Te-aug-me_1.jpg Meningioma	Te-no_1.jpg Notumor	Te-pi_1.jpg Pituitary
No Attack	✔	✔	✔	✔
Horizontal Flip	×	✔	✔	✔
Affine Transform	✔	✔	✔	✔
Rotation	×	✔	✔	✔
Vertical Flip	✔	✔	✔	✔
Gaussian Blur	×	✔	✔	×
Speckle Noise	✔	✔	✔	✔
Motion Blur	×	✔	✔	✔
Contrast Shift	×	✔	✔	✔
Saturation Shift	✔	✔	✔	✔
Brightness Shift	×	✔	✔	✔
Gaussian Noise	×	✔	✔	✔
JPEG Compression	×	✔	✔	✔
Random Erasing	✔	✔	✔	✔
Hue Shift	✔	✔	✔	✔

**Table 20:** Trends in classification performance of the proposed model under various test-time perturbations.

Attack	Accuracy (%)	AUC-ROC	Specificity	TP	TN	FP	FN	Acc_Drop (%)
No Attack	95.438	0.986	0.985	1527	4727	73	73	0
Saturation Shift	95.438	0.986	0.985	1527	4727	73	73	0
Hue Shift	95.438	0.986	0.985	1527	4727	73	73	0
Horizontal Flip	95.250	0.986	0.984	1524	4724	76	76	0.188
Affine Transform	95.250	0.986	0.984	1524	4724	76	76	0.188
JPEG Compression	95.250	0.984	0.984	1524	4724	76	76	0.188
Speckle Noise	95.000	0.985	0.983	1520	4720	80	80	0.438
Rotation	94.438	0.983	0.981	1511	4711	89	89	1.000
Brightness Shift	94.313	0.980	0.981	1509	4709	91	91	1.125
Random Erasing	92.313	0.975	0.974	1477	4677	123	123	3.125
Motion Blur	91.250	0.975	0.971	1460	4660	140	140	4.188
Contrast Shift	90.813	0.977	0.969	1453	4653	147	147	4.625
Gaussian Noise	90.250	0.978	0.968	1444	4644	156	156	5.188
Gaussian Blur	84.875	0.969	0.950	1358	4558	242	242	10.563
Vertical Flip	81.625	0.965	0.939	1306	4506	294	294	13.813

**Table 21:** Near-duplicate check between training and test splits using perceptual hashing.

Class	N_Train	N_Test	Exact Duplicates	Near Duplicates	Flagged Rate (%)
Glioma	1400	400	0	358	89.5
Meningioma	1400	400	0	392	98.0
No Tumor	1400	400	0	396	99.0
Pituitary	1400	400	0	366	91.5
Total	5600	1600	0	1512	94.5

## Data Availability

The original datasets presented in the study are openly available on Kaggle at Brain Tumor MRI Dataset. https://www.kaggle.com/datasets/masoudnickparvar/brain-tumor-mri-dataset (accessed on 27 May 2026).
